# Selectivity Control in Nitroaldol (Henry) Reaction by Changing the Basic Anion in a Chiral Copper(II) Complex Based on (*S*)-2-Aminomethylpyrrolidine and 3,5-Di-*tert*-butylsalicylaldehyde

**DOI:** 10.3390/molecules29215207

**Published:** 2024-11-04

**Authors:** Olga V. Khromova, Lidiya V. Yashkina, Nadezhda V. Stoletova, Victor I. Maleev, Yuri N. Belokon, Vladimir A. Larionov

**Affiliations:** 1 A.N. Nesmeyanov Institute of Organoelement Compounds of Russian Academy of Sciences (INEOS RAS), Vavilov Str. 28, Bld. 1, 119334 Moscow, Russia; olvichrom@ineos.ac.ru (O.V.K.); stoletovanv@gmail.com (N.V.S.); vim@ineos.ac.ru (V.I.M.); 2 Faculty of Science, Peoples’ Friendship University of Russia (RUDN University), Miklukho-Maklaya Str. 6, 117198 Moscow, Russia

**Keywords:** henry reaction, copper complex, nitroalcohols, nitrostyrenes, enantioselectivity

## Abstract

This article is a continuation of our previous research on the catalytic capability of a chiral copper complex based on commercially available (*S*)-2-aminomethylpyrrolidine and 3,5-di-*tert*-butylsalicylaldehyde with various counter-anions in the asymmetric Henry reaction. Our findings indicate that depending on the type of base used, chiral nitroalcohols with yields up to 98% and *ee* values up to 77%, as well as β-nitrostyrenes with yields up to 88%, can be produced. Additionally, it has been found that the outcome of the reaction and the catalytic properties of copper (II) complexes (*S*)-**Cu1** and (*S*)-**Cu2** are influenced by the structure of the aldehyde used.

## 1. Introduction

**Scheme 1:**
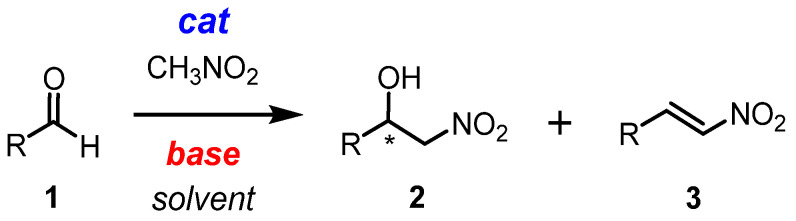
Henry (nitroaldol) reaction. * It is used to mark the stereocenter.

The nitroaldol reaction, also known as the Henry reaction [1,2], is a classical and versatile method for producing chiral β-nitroalcohols **2** (Scheme 1) [3,4,5,6,7,8]. These compounds can then be converted into a variety of valuable products, such as α-nitroketones [9], nitroalkenes [10,11,12,13], β-aminoalcohols [14], and alkanes. They can even be used to produce some pharmaceuticals [15,16,17,18,19]. Various catalytic systems have been employed for this reaction, including the chiral catalysts [20,21,22,23,24,25,26,27,28,29,30,31,32,33,34,35,36,37,38,39,40,41,42,43,44,45,46,47,48,49]. In most cases, a combination of both Lewis acid and Brønsted base is necessary for efficient catalysis. Obviously, it is a challenge to combine both of these aspects in the same system. Depending on the balance of the Brønsted basicity and Lewis acidity of the catalyst, the reaction may also produce dehydration products such as nitroalkenes **3**, which are also important building blocks in organic synthesis (Scheme 1) [50,51,52,53,54,55]. The control of selectivity in the nitroaldol reaction and its mechanistic study has not been a major focus of research in this field. However, the nature of the basic anion used in the catalyst may determine whether the reaction produces β-nitroalcohols or β-nitrostyrenes. By controlling the selectivity through a simple change in the basicity of the catalytic system via anion exchange, it is possible to produce enantiomerically enriched nitroalcohols or nitrostyrenes while making minimal changes to the reaction conditions. This is of significant interest for synthetic applications.

Despite the importance of nitroalkenes **3** as diverse building blocks, there have been only a few reports so far on their formation when using copper complexes as catalysts [56,57,58,59,60,61,62]. For example, Luo and Yan used a homogenous copper(II) complex based on chiral α-ethylphenylamines in the asymmetric Henry reaction, and obtained accompanied nitrostyrene products with up to 47% yield under the reaction conditions [56]. The Jones and Schulz groups also showed that immobilizing chiral copper(II) complexes on a solid support led to the formation of nitroalkenes **3** (up to 35%) [57,58]. This is likely due to the intrinsic acidity of the silica-based support. However, systematic and in-depth studies on the influence of the ligand Brønsted basicity and Lewis acidity of the central metal cations and the nature of the anion on selectivity in the aldol condensation have not been undertaken.

**Figure 1:**
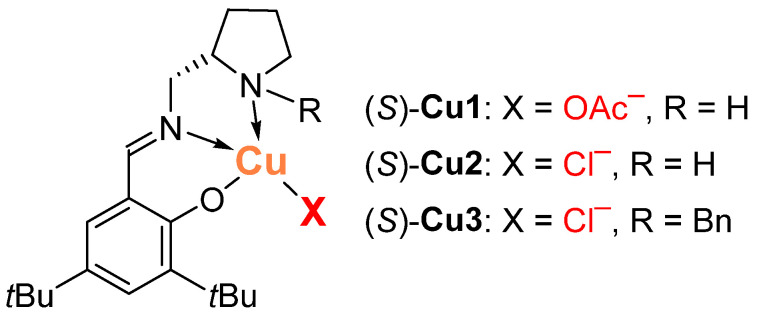
Chiral copper(II) complexes **Cu1**–**Cu3**.

Here, we set out to partially fill this knowledge gap by using chiral copper(II) complexes that we had previously developed [63,64], derived from the tridentate Schiff base of (*S*)-2-aminomethylpyrrolidine and 3,5-di-*tert*-butylsalicylaldehyde and different basic anions (Figure 1) in the reaction of nitromethane with various aldehydes. We hypothesized that by varying the nature of the basic anion in the complex, we can change the selectivity of the reaction and obtain either β-nitroalcohol **2** or nitroalkene **3**, which are both valuable building blocks in synthetic chemistry [3,4,5,6,7,8,9,10,11,12,13,14,15,16,17,18,19].

## 2. Results and Discussion

### 2.1. Screening of Henry Reaction Conditions 

We previously demonstrated that the chiral copper(II) complex (*S*)-**Cu1** catalyzes the Henry reaction of *o*-nitrobenzaldehyde **1a** with nitromethane, producing the nitroalcohol **2a** in 78% yield with 77% *ee* (Table 1, entry 1) [64]. On the other hand, when the temperature was increased to 50 °C, nitrostyrene **3a** formed in 55% yield and the enantiomeric purity of **2a** also dropped to 25% (Table 1, entry 2). To improve the yield, an additional quantity of acetate base was used, resulting in a significant increase in the yield of **2a** to 96%, with an *ee* of 73% (Table 1, entry 4). When the reaction time was extended to 65 h, a similar yield of 92% was obtained, along with 7% of nitrostyrene being formed, but with a decrease in *ee* to 55% (Table 1, entry 5). This may be attributed to a partial racemization process.

As expected, the complex (*S*)-**Cu2** with a non-basic chloride anion was found to be catalytically inactive (Table 1, entry 7). Therefore, the next step was to investigate the basicity of the anion in the copper(II) complex. The in situ exchange of the chloride anion with a strong basic phenolate accelerated the conversion, leading to the formation of 53% of **2a** and 40% of compound **3a** after 3 h (Table 1, entry 9). Similar results were obtained with another strong basic anion (O^2−^), which was generated in situ using Ag_2_O (Table 1, entry 11). Increasing the reaction time led to a higher proportion of compound **3a** compared to **2a** formed (Table 1, entry 12). The reaction performed with deuterated nitromethane gave product **3a** with a lower yield (24%) (Table 1; compare entries 14 and 15).

The nature of the solvent played an essential role in the reaction, significantly influencing the outcome (Table 2). The highest yields of product **3a** were achieved when using 1,2-dichloroethane (DCE) and toluene, with yields of 63% and 61%, respectively (Table 2, entries 1 and 6). The reaction, proceeding without a solvent, with 30 equivalents of nitromethane produced nitroalcohol **2a** with 44% yield and nitrostyrene **3a** with 35% yield. Additionally, Michael addition product **4a** was formed in 20% yield (Table 2, entry 8). It is worth noting that the reaction in methanol using the catalytic system (*S*)-**Cu2**/Ag_2_O resulted in only nitroalcohol **2a** in a quantitative yield (99%), but in a racemic form (Table 2, entry 7).

Next, the impact of temperature on the reaction outcome was studied (Table 1 and Table 3). Lowering the temperature to −17 °C increased the yield of product **2a** (98% for PhONa and 89% for Ag_2_O), with a trace amount of **3a** forming; however, the enantioselectivity of the reaction was decreased (Table 1, entries 10 and 13). On the other hand, when the reaction was performed using complex (*S*)-**Cu1** at −17 °C, the enantioselectivity did not change (Table 1; compare entries 4 and 6). Conversely, when the reaction was carried out at 50 °C, the yield of product **3a** increased to 81% (Table 3, entry 1). Further increasing the temperature to 70 °C yielded the desired β-nitrostyrene **3a** in 81%, although the yield of *bis*-coupled product **4a** rose to 18% (Table 3, entry 2).

Then, the decrease in catalyst and MeNO_2_ loadings was investigated (Table 3, entries 3–5). The maximum yield of nitrostyrene **3a** (88%) was obtained with 2 mol% of the complex (*S*)-**Cu2** and 1 mol% of Ag_2_O and 5 equivalents of MeNO_2_ used (Table 3, entry 5). The *N*-benzylated complex (*S*)-**Cu3** influenced the outcome of the reaction, resulting in only 11% of nitroalcohol **2a** being produced in the presence of NaOAc at room temperature (Table 3, entry 6), whereas the increase in temperature to 70 °C yielded nitroalcohol **2a** with 6% yield and nitrostyrene **3a** as the main product (75%), accompanied by 17% of compound **4a** (Table 3, entry 8).

For comparison purposes, the reaction using the catalytic system CuCl_2_/1,10-phenanthroline/Ag_2_O was studied under optimized conditions, and the yield of **3a** was only 23% as compared to 88% in the case of (*S*)-**Cu2** (Table 3, entry 9). It should be noted that pure silver oxide does not catalyze the reaction on its own (Table 3, entry 10). In contrast, the reaction catalyzed by *t*BuOK resulted in the formation of only nitroalcohol **2a** with 85% conversion (Table 3, entry 11).

This demonstrates the superiority of our new catalytic system, the selectivity of which can be easily modified by the simple modification of the tridentate ligand or the nature of the mono-anion.

### 2.2. The Scope of Aldehydes in the Enantioselective Henry Reaction with the Complex (S)-**Cu1**

**Scheme 2:**
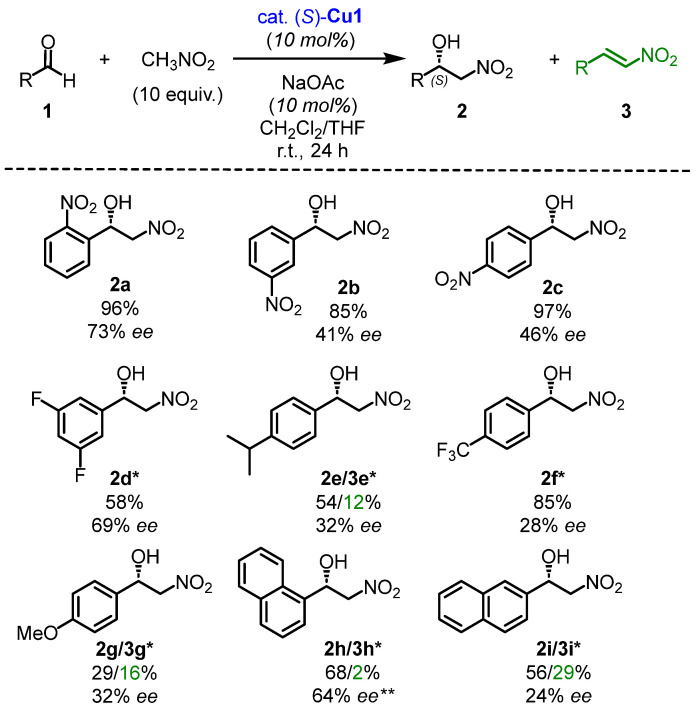
Henry reaction catalyzed by the complex (*S*)-**Cu1**. Reaction conditions: aldehyde **1** (0.3 mmol), nitromethane (10 equiv., 3 mmol), complex (*S*)-**Cu1** (10 mol%), and NaOAc (10 mol%) in 0.5 mL of solvent mixture (CH_2_Cl_2_/THF) were stirred for 24 h. The yields were determined by the ^1^H NMR analysis of the crude mixture using HMDSO as a standard. * The reaction time was 36 h. ** No base was used.

The next step was to examine the reactivity of various aldehydes in the enantioselective Henry reaction catalyzed by (*S*)-**Cu1** (Scheme 2). Experiments showed that the substituents on the aromatic rings of aldehydes significantly affected the yields of products **2** and **3**. The catalyst (*S*)-**Cu1** demonstrated good activity with benzaldehyde with an electron-withdrawing group (EWG). *Ortho*-, *meta*-, and *para*-NO_2_-substituted nitroalcohols **2a**–**2c** were produced in high yields (85–97%) with no traces of nitrostyrene. A similar trend was observed for benzaldehydes substituted with fluorine, resulting in **2d** with 58% yield and **2f** with 85% yield, respectively. However, for aldehydes with electron-donating groups (EDGs), the targeted Henry reaction occurred along with the dehydration process. The resulting products **2e** and **2g**–**2i** were isolated with moderate yields (54%, 29%, 68%, and 56%, respectively), accompanied by the formation of nitrostyrenes **3e** and **3g**–**3i** with 12%, 16%, 2%, and 29% yields, respectively. The level of enantioselectivity for the nitroalcohols **2a**–**2i** was also moderate, ranging from 24 to 73% *ee*.

### 2.3. The Scope of Aldehydes in the Synthesis of β-Nitrostyrenes ***3***

**Scheme 3:**
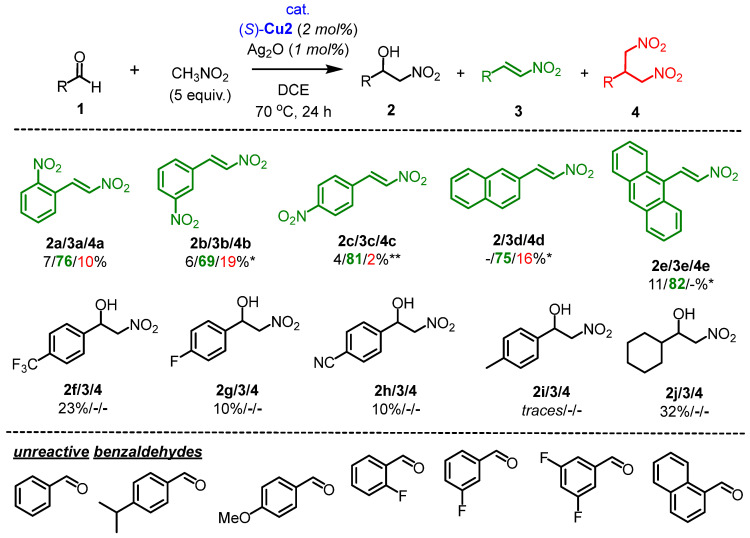
Henry reaction catalyzed by a catalytic system (*S*)-**Cu2**/Ag_2_O. Reaction conditions: aldehyde **1** (0.3 mmol), nitromethane (5 equiv., 1.5 mmol), complex (*S*)-**Cu2** (2 mol%), and Ag_2_O (1 mol%) in 1 mL of DCE were stirred for 24 h at 70 °C. * (*S*)-**Cu2** (5 mol%), Ag_2_O (2.5 mol%), CH_3_NO_2_ 10 equiv. ** The reaction time was 48 h.

Next, the nitroaldol reaction was extended using various aldehydes in the presence of a catalytic system consisting of (*S*)-**Cu2**/Ag_2_O, which favored the formation of nitrostyrene **3** (Scheme 3). The selectivity and efficiency of this catalytic system were found to be highly dependent on the structure of **1**. The reaction showed a high conversion rate for aldehydes **1a**–**1e**, with nitrostyrene **3** being the predominant product (*o*-NO_2_ **3a**: 76%, *m*-NO_2_ **3b**: 69%, *p*-NO_2_ **3c**: 81%, 2-naphthyl **3d**: 75% and 9-anthracenyl **3e**: 82%, respectively) (Scheme 3). It is worth noting that the corresponding *trans*-β-nitrostyrenes **3a**-**3e** were isolated in high purity through crystallization with yields ranging from 58% to 74%. However, for some aldehydes (**1f**–**1j**), depicted in Scheme 3, the reaction did not proceed efficiently, resulting in low conversions and the formation of nitroalcohols in up to 32% yield. Most likely, the rate of the retro-nitroaldol reaction was faster as compared to the irreversible dehydration step (vide infra). Therefore, it seems reasonable to suggest that the (*S*)-**Cu2** complex requires particularly strong EWGs in the benzaldehyde structure (such as NO_2_) to achieve efficient catalysis. Even with CF_3_ and CN groups, the catalytic activity of (*S*)-**Cu2** is already limited.

### 2.4. Mechanism-Related Experiments

**Scheme 4:**
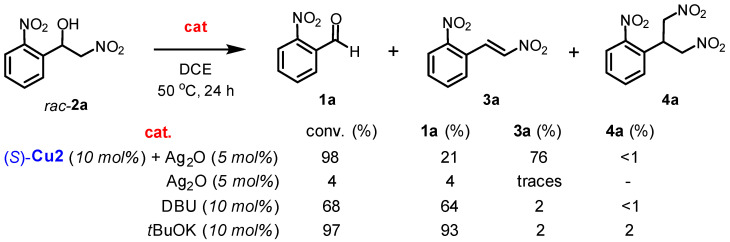
Control experiments.

In order to understand the mechanism of the addition of nitromethane to benzaldehydes and the subsequent dehydration process, a series of retro-nitroaldol experiments were conducted under condensation conditions (Scheme 4). Racemic nitroalcohol **2a** was dissolved in a solution of DCE with *t*BuOK as a strong base for 24 h at 50 °C. This led to the complete decomposition of nitroalcohol into aldehyde **1a** (97% conversion). Similar results were obtained when DBU was used as an organic base, although the conversion was incomplete (68%). Silver oxide did not decompose nitroalcohol or promote dehydration process. As expected, the combined system of (*S*)-**Cu2**/Ag_2_O produced a high yield of nitrostyrene **3a** (76%), along with the initial aldehyde **1a** in 21% yield (Scheme 4).

**Figure 2:**
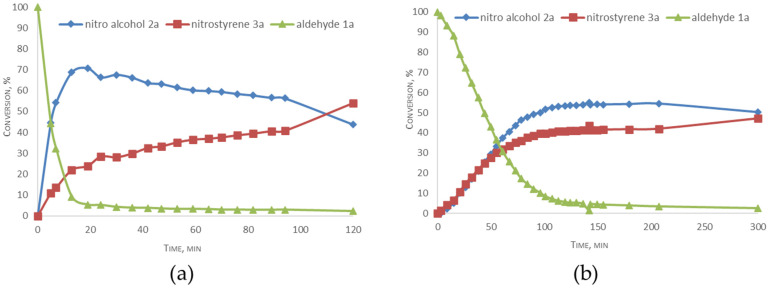
Kinetic profiles of the reaction with nitromethane (**a**) and deuterated nitromethane (**b**) in CD_2_Cl_2_.

^1^H NMR kinetic studies of the condensation reaction between benzaldehyde **1a** and CH_3_NO_2_ and deuterated CD_3_NO_2_ in CD_2_Cl_2_ were also conducted (Figure 2). According to the data, the aldol condensation step of the reaction showed a kinetic isotope effect (k_D_/k_H_) of approximately 10 and 3 for the dehydration process. This result corroborates the involvement of C-D bond cleavage in the rate-limiting stages of both reactions.

**Scheme 5:**
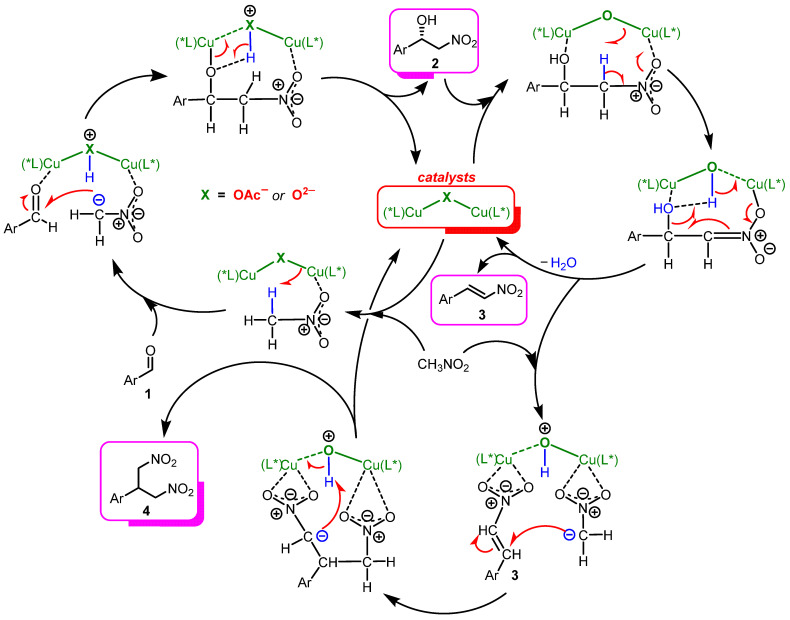
Proposed mechanism for the Henry and subsequent reactions catalyzed by (*S*)-**Cu1** or by the system (*S*)-**Cu2** and Ag_2_O. (*L) in the Scheme refers to a chiral ligand based on (*S*)-(2-aminomethyl)pyrrolidine and 3,5-di-*tert*-butylsalicylaldehyde.

Finally, based on the experimental results and findings, we propose a mechanism for the catalysis (Scheme 5). The first step is the formation of a catalytically active dimeric complex from (*S*)-**Cu2** and Ag_2_O, where two units of copper are linked by a μ^2^-oxygen bridge ([L-Cu]-O-[L-Cu]) [65]. This catalytic particle with a higher basicity of the oxygen bridge, promotes the reversible nitroaldol reaction, leading to the formation of nitroalcohol **2**. Subsequently, the dehydration process is accelerated as the complex [L-Cu]-O-[L-Cu] appears to coordinate with product **2**, increasing the acidity of its β-protons and activating the leaving hydroxyl groups of nitroalcohols by coordinating with the Cu ion, thus facilitating the E1cB elimination step for the production of β-nitrostyrene **3** (Scheme 5). This concept explains why strongly basic *t*BuOK promotes only the retro-aldol decomposition of nitroalcohols and not their dehydration. It should also be noted that the Michael addition reaction of the second nitromethane molecule leads to the formation of a minor *bis*-coupled product **4** [66].

## 3. Materials and Methods

### 3.1. General Information

All solvents purchased from commercial suppliers were used without further purification (CH_2_Cl_2_, DCE, MeOH, 1,4-dioxane, CDCl_3_, CD_2_Cl_2_, and acetone-*d*_6_). THF and toluene were distilled over sodium under an atmosphere of argon. (*S*)-(2-aminomethyl)pyrrolidine, 3,5-di-*tert*-butylsalicylaldehyde, nitromethane, DBU, CD_3_NO_2_, *t*BuOK, NaOAc, Ag_2_O, and Cu(OAc)_2_ purchased from commercial suppliers were used without further purification. Commercially available benzaldehydes were purified by distillation under reduced pressure or through flash SiO_2_ column. (*S*)-2-aminomethyl-1-benzylpyrrolidine was synthesized according to a procedure in the literature, starting from 1-benzyl-(*S*)-proline [67]. The (*S*)-**Cu1**, (*S*)-**Cu2**, and (*S*)-**Cu3** complexes were synthesized according to our previously published procedure [63,64]. The reported catalytic reactions were performed under an atmosphere of argon using Schlenk-line techniques in flame-dried glassware. Unless stated otherwise, flash column chromatography was performed with silica gel 60 M from Macherey-Nagel.

### 3.2. Instrumentation

Proton nuclear magnetic resonance (^1^H NMR) spectra were recorded on a Bruker Avance 300 NMR spectrometer (operating at 300 MHz, referring to ^1^H nucleus). Chemical shifts are reported in ppm relative to the residual solvent peak (CDCl_3_: *δ* = 7.26 ppm for ^1^H NMR). NMR data are reported as follows: chemical shift, multiplicity (br. s = broad singlet; s = singlet; d = doublet; dd = doublet of doublets; m = multiplet), coupling constant, integration, and nucleus (Supplementary Materials). Chiral HPLC chromatography was performed with a Shimadzu LC-10ADVP instrument equipped with a Shimadzu SPDM10AVP diode array detector using a Chiralcel OD-H column (conditions: hexane/isopropanol = 90:10 or 95:5; flow rate: 1 mL/min; 254 nm; 25 °C).

### 3.3. Synthesis

#### 3.3.1. General Procedure for the Racemic Henry Reaction

A flask (5 mL) was charged with aldehyde **1** (0.3 mmol, 1.0 equiv.), DBU (4.5 µL, 10 mol%), and DCM (0.5 mL) under Ar atmosphere. Then, nitromethane (0.16 mL, 3 mmol, 10 equiv.) was added and the reaction mixture was stirred for 24 h at RT. After the reaction was complete, the mixture was purified by flash SiO_2_ chromatography (eluent: CH_2_Cl_2_). The solvent was evaporated on a rotary evaporator, and the residue was used as a racemic standard for HPLC analysis without further purification.

#### 3.3.2. General Procedure for the Enantioselective Henry Reaction

A flask (10 mL) was charged with aldehyde **1** (0.3 mmol, 1 equiv.), (*S*)-**Cu1** catalyst (13.2 mg, 10 mol %), and NaOAc (2.4 mg, 10 mol %) under Ar atmosphere. Then, a solvent mixture of THF/DCM (0.25 mL/0.25 mL) and nitromethane (0.160 mL, 3 mmol, 10 equiv.) was added and the reaction mixture was stirred for 24 h at RT. After the reaction was complete, the reaction mixture was purified by flash SiO_2_ chromatography (eluent: CH_2_Cl_2_). The solvent was evaporated on a rotary evaporator, and the residue was purified by column SiO_2_ chromatography (eluent: hexane/acetone (5:1)) to afford the desired (*S*)-product **2**.

1-(2-nitrophenyl)-2-nitroethan-1-ol (**2a**)
^1^H NMR (400 MHz, CDCl_3_): δ = 8.11–8.08 (m, 1H, ArH), 7.98–7.96 (m, 1H, ArH), 7.78–7.74 (m, 1H, ArH), 7.59–7.55 (m, 1H, ArH), 6.07 (ddd, J = 8.8, 4.2, 2.2 Hz, 1H), 4.89 (dd, J = 13.9, 2.2 Hz, 1H), 4.57 (dd, J = 13.9, 8.8 Hz, 1H), 3.15 (d, J = 4.2 Hz, 1H) ppm.
All spectroscopic data were in agreement with the literature [64].
The enantiomeric excess was established by HPLC analysis using a Kromasil 3-AmyCoat column, *ee* = 73% (conditions: heptane/isopropanol = 90:10; flow rate: 1 mL/min; 254 nm; 25 °C; t_R_(major) = 12.7 min; t_R_(minor) = 11.0 min).
All spectroscopic data were in agreement with the literature [63].
1-(3-nitrophenyl)-2-nitroethan-1-ol (**2b**)
^1^H NMR (CDCl_3_, 300 MHz): δ = 8.33–8.28 (m, 1H), 8.24–8.15 (m, 1H), 7.77 (d, J = 7.7 Hz, 1H), 7.64–7.56 (m, 1H), 5.66–5.55 (m, 1H), 4.68–4.54 (m, 2H), 3.46–3.40 (m, 1H) ppm.
The enantiomeric excess was established by HPLC analysis using a Chiralcel OD-H column, *ee* = 41% (conditions: hexane/isopropanol = 90:10; flow rate: 1 mL/min; 254 nm; 25 °C; t_R_(major) = 28.4 min; t_R_(minor) = 25.0 min).
All spectroscopic data were in agreement with the literature [68].
1-(4-nitrophenyl)-2-nitroethan-1-ol (**2c**)
^1^H NMR (CDCl_3_, 300 MHz): δ = 8.26 (d, J = 8.7 Hz, 2H), 7.62 (d, J = 8.6 Hz, 2H), 5.65–5.56 (m, 1H), 4.67–4.51 (m, 2H), 3.33–3.26 (m, 1H) ppm.
The enantiomeric excess was established by HPLC analysis using a Chiralcel OD-H column, *ee* = 46% (conditions: hexane/isopropanol = 90:10; flow rate: 1 mL/min; 254 nm; 25 °C; t_R_(major) = 31.4 min; t_R_(minor) = 25.3 min).
All spectroscopic data were in agreement with the literature [68].
1-(3,5-difluorophenyl)-2-nitroethan-1-ol (**2d**)
^1^H NMR (CDCl_3_, 300 MHz): δ = 7.02–6.91 (m, 2H), 6.85–6.74 (m, 1H), 5.51–5.41 (m, 1H), 4.62–4.46 (m, 2H), 3.14–3.07 (m, 1H) ppm. ^19^F NMR (282 MHz, CDCl_3_): δ = −107.7 (s, 2F) ppm.
The enantiomeric excess was established by HPLC analysis using a Chiralcel OD-H column, *ee* = 69% (conditions: hexane/isopropanol = 90:10; flow rate: 1 mL/min; 254 nm; 25 °C; t_R_(major) = 12.5 min; t_R_(minor) = 10.7 min).
All spectroscopic data were in agreement with the literature [23].
1-(4-isopropylphenyl)-2-nitroethan-1-ol (**2e**)
^1^H NMR (CDCl_3_, 300 MHz): δ = 7.36–7.30 (m, 2H), 7.29–7.23 (m, 2H), 5.48–5.39 (m, 1H), 4.61 (dd, J = 13.3, 9.6 Hz, 1H), 4.50 (dd, J = 13.2, 3.1 Hz, 1H), 3.00–2.86 (m, 1H), 1.25 (d, J = 6.9 Hz, 6H) ppm.
The enantiomeric excess was established by HPLC analysis using a Chiralcel OD-H column, *ee* = 32% (conditions: hexane/isopropanol = 90:10; flow rate: 1 mL/min; 254 nm; 25 °C; t_R_(major) = 18.2 min; t_R_(minor) = 12.2 min).
All spectroscopic data were in agreement with the literature [69].
1-(4-(trifluoromethyl)phenyl)-2-nitroethan-1-ol (**2f**)
^1^H NMR (CDCl_3_, 300 MHz): δ = 7.67 (d, J = 8.1 Hz, 2H), 7.55 (d, J = 8.1 Hz, 2H), 5.60–5.48 (m, 1H), 4.65–4.48 (m, 2H), 3.21–3.09 (m, 1H) ppm. ^19^F NMR (282 MHz, CDCl_3_): δ = −62.7 (s, 3F) ppm.
The enantiomeric excess was established by HPLC analysis using a Chiralcel OD-H column, *ee* = 28% (conditions: hexane/isopropanol = 90:10; flow rate: 1 mL/min; 254 nm; 25 °C; t_R_(major) = 14.6 min; t_R_(minor) = 11.5 min).
All spectroscopic data were in agreement with the literature [68].
1-(4-methoxyphenyl)-2-nitroethan-1-ol (**2g**)
^1^H NMR (CDCl_3_, 300 MHz): δ = 7.35–7.28 (m, 2H), 6.95–6.89 (m, 2H), 5.45–5.37 (m, 1H), 4.60 (dd, J = 13.2, 9.6 Hz, 1H), 4.47 (dd, J = 13.2, 3.1 Hz, 1H), 3.81 (s, 3H), 2.84–2.80 (m, 1H) ppm.
The enantiomeric excess was established by HPLC analysis using a Chiralcel OD-H column, *ee* = 32% (conditions: hexane/isopropanol = 90:10; flow rate: 1 mL/min; 254 nm; 25 °C; t_R_(major) = 26.0 min; t_R_(minor) = 20.3 min).
All spectroscopic data were in agreement with the literature [68].
1-(naphthalen-1-yl)-2-nitroethan-1-ol (**2h**)
^1^H NMR (CDCl_3_, 300 MHz): δ = 8.04 (d, J = 8.3 Hz, 1H), 7.94–7.89 (m, 1H), 7.89–7.83 (m, 1H), 7.77 (d, J = 7.2 Hz, 1H), 7.64–7.49 (m, 3H), 6.30–6.23 (m, 1H), 4.74–4.59 (m, 2H), 2.92 (d, J = 3.6 Hz, 1H) ppm.
The enantiomeric excess was established by HPLC analysis using a Chiralcel OD-H column, *ee* = 64% (conditions: hexane/isopropanol = 90:10; flow rate: 1 mL/min; 254 nm; 25 °C; t_R_(major) = 24.8 min; t_R_(minor) = 18.0 min).
All spectroscopic data were in agreement with the literature [69].
1-(naphthalen-2-yl)-2-nitroethan-1-ol (**2i**)
^1^H NMR (CDCl_3_, 300 MHz): δ = 7.90–7.81 (m, 4H), 7.57–7.49 (m, 2H), 7.49–7.41 (m, 1H), 5.65–5.56 (m, 1H), 4.68 (dd, J = 13.3, 9.4 Hz, 1H), 4.57 (dd, J = 13.3, 3.2 Hz, 1H), 3.16–3.10 (br. s, 1H) ppm.
The enantiomeric excess was established by HPLC analysis using a Chiralcel OD-H column, *ee* = 24% (conditions: hexane/isopropanol = 90:10; flow rate: 1 mL/min; 254 nm; 25 °C; t_R_(major) = 52.5 min; t_R_(minor) = 37.1 min).
All spectroscopic data were in agreement with the literature [23].

#### 3.3.3. General Procedure for the Synthesis of β-Nitrostyrenes **3**

A flask (10 mL) was charged with aldehyde **1** (0.3 mmol, 1 equiv.), complex (*S*)-**Cu2** (2.4 mg, 2 mol% or 6.2 mg, 5 mol%). and Ag_2_O (0.7 mg, 1 mol% or 1.8 mg, 2.5 mol%). Then, 1,2-dichloroethane (1 mL) and nitromethane (0.08 mL, 1.5 mmol, 5 equiv. or 0.16 mL, 3.0 mmol, 10 equiv.) were added and the reaction mixture was stirred for 24 h at 70 °C. After the reaction was complete, the reaction mixture was filtrated through the SiO_2_ layer using CH_2_Cl_2_ as an eluent. The solvent was evaporated on a rotary evaporator, and the residue was purified by column SiO_2_ chromatography or by crystallization in ethanol to afford the desired product **3**.

2-nitro-β-nitrostyrene (**3a**)
^1^H NMR (300 MHz, CDCl_3_): δ = 8.54 (d, J = 13.4 Hz, 1H), 8.21 (d, J = 7.9 Hz, 1H), 7.81–7.65 (m, 2H), 7.61 (d, J = 7.3 Hz, 1H), 7.43 (d, J = 13.5 Hz, 1H) ppm.
All spectroscopic data were in agreement with the literature [70].
3-nitro-β-nitrostyrene (**3b**)
^1^H NMR (300 MHz, CDCl_3_): δ = 8.42 (t, J = 2.0 Hz, 1H), 8.35 (dd, J = 8.4, 2.2 Hz, 1H), 8.06 (d, J = 13.7 Hz, 1H), 7.88 (d, J = 7.7 Hz, 1H), 7.74–7.63 (m, 2H) ppm.
All spectroscopic data were in agreement with the literature [71].
4-nitro-β-nitrostyrene (**3c**)
^1^H NMR (300 MHz, acetone-d_6_): δ = 8.33 (d, J = 8.8 Hz, 2H), 8.25–8.07 (m, 4H) ppm.
All spectroscopic data were in agreement with the literature [70].
(E)-2-(2-nitrovinyl)naphthalene (**3d**)
^1^H NMR (300 MHz, CDCl_3_): δ = 8.15 (d, J = 13.6 Hz, 1H), 8.00 (s, 1H), 7.88 (dt, J = 9.4, 3.6 Hz, 3H), 7.69 (d, J = 13.6 Hz, 1H), 7.65–7.51 (m, 3H) ppm. ^13^C NMR (101 MHz, CDCl_3_): δ = 139.4, 137.3, 135.0, 133.3, 132.4, 129.5, 129.0, 128.5, 128.1, 127.7, 127.4, 123.4 ppm.
(E)-9-(2-nitrovinyl)anthracene (**3e**)
^1^H NMR (300 MHz, CDCl_3_): δ = 8.98 (d, J = 13.7 Hz, 1H), 8.53 (s, 1H), 8.11 (dd, J = 39.5, 8.4 Hz, 4H), 7.76–7.39 (m, 5H) ppm. ^13^C NMR (101 MHz, CDCl_3_): δ = 142.8, 135.8, 131.2, 130.6, 130.0, 129.3, 127.6, 125.8, 124.4, 123.3 ppm.

## 4. Conclusions

In conclusion, we have demonstrated that a chiral copper(II) complex derived from a Schiff base ligand based on commercially available (*S*)-2-aminomethylpyrrolidine and 3,5-di-*tert*-butylsalicylaldehyde can catalyze the Henry reaction selectively, depending on the type of anion present. The chiral complex containing acetate as the anion allows for the formation of nitroalcohols with high yields, up to 97%, and *ee* values up to 77%, while the in situ formed catalytic system [L-Cu]-O-[L-Cu] predominantly produces β-nitrostyrenes with yields up to 88%. Additionally, the substituents on the aromatic rings of aldehydes significantly impact the reaction outcomes, leading to the formation of different nitroolefins. Our findings clearly show that the selectivity of the copper(II) complex can be readily adjusted by modifying the ligand structure and the basicity of the anion.

## Figures and Tables

**Table 1:** Screening of reaction conditions for the nitroaldol reaction of **1a** *^a^*.

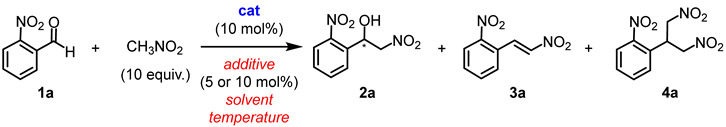							
Entry	Catalyst	Additive (*x* mol%)	Solvent	T, °C	Yield (*ee*) of 2a (%) *^b^*^,*c*^	Yield of 3a (%) *^b^*	Yield of 4a (%) *^b^*
1 [64]	(*S*)-**Cu1**	–	CD_2_Cl_2_ *^d^*	RT	78 (77)	–	–
2	(*S*)-**Cu1**	–	DCE	50	43 (25)	55	traces
3 [64]	(*S*)-**Cu1**	–	THF/CH_2_Cl_2_ (1:1)	RT	47 (76)	–	–
4	(*S*)-**Cu1**	NaOAc (10)	THF/CH_2_Cl_2_ (1:1)	RT	96 (73)	2	–
5 *^e^*	(*S*)-**Cu1**	NaOAc (10)	THF/CH_2_Cl_2_ (1:1)	RT	92 (55)	7	–
6 *^e^*	(*S*)-**Cu1**	NaOAc (10)	THF/CH_2_Cl_2_ (1:1)	−17	92 (73)	traces	–
7	(*S*)-**Cu2**	–	CH_2_Cl_2_	RT	NR	–	–
8 [64]	(*S*)-**Cu2**	NaOAc (10)	THF/CH_2_Cl_2_ (1:1)	RT	96 (77)	–	–
9 *^f^*	(*S*)-**Cu2**	PhONa (10)	CH_2_Cl_2_	RT	53 (12)	40	3
10	(*S*)-**Cu2**	PhONa (10)	CH_2_Cl_2_	−17	98 (39)	traces	Traces
11 *^f^*	(*S*)-**Cu2**	Ag_2_O (5)	CH_2_Cl_2_	RT	59 (0)	34	2
12	(*S*)-**Cu2**	Ag_2_O (5)	CH_2_Cl_2_	RT	31 (ND)	54	14
13	(*S*)-**Cu2**	Ag_2_O (5)	CH_2_Cl_2_	−17	89 (56)	4	Traces
14 *^f^*^,*g*^	(*S*)-**Cu2**	Ag_2_O (5)	CH_2_Cl_2_	RT	71	24	Traces
15 *^g^*	(*S*)-**Cu2**	Ag_2_O (5)	CH_2_Cl_2_	RT	50	48	2

*^a^* Reaction conditions: *o*-nitrobenzaldehyde **1a** (0.15 mmol), nitromethane (10 equiv., 1.5 mmol), catalyst (10 mol%), and additive (5 or 10 mol%) in 0.5 mL solvent were stirred for 24 h. *^b^* The yields were determined by the ^1^H NMR analysis of the crude mixture. *^c^* Enantiomeric excess was determined by chiral HPLC analysis. *^d^* 0.1 equiv. of water was added. *^e^* The reaction time was 65 h. *^f^* The reaction time was 3 h. *^g^* CD_3_NO_2_ was used instead of CH_3_NO_2_. DCM = dichloromethane. DCE = 1,2-dichloroethane. THF = tetrahydrofuran. NR = no reaction. ND = not determined.

**Table 2:** The solvent screening for the nitroaldol reaction of **1a** catalyzed by (*S*)-**Cu2** and Ag_2_O *^a^*.

Entry	1	2	3	4	5	6	7	8
**solvent**	DCE	CH_3_CN	EtOAc	THF	1,4-dioxane	toluene	MeOH	CH_3_NO_2_ *^c^*
**Yield of 2a (%) *^b^***	23	72	51	69	51	28	99 *^d^*	44
**Yield of 3a (%) *^b^***	63	16	30	15	32	61	–	35
**Yield of 4a (%) *^b^***	13	11	18	15	16	10	–	20

*^a^* Reaction conditions: *o*-nitrobenzaldehyde **1a** (0.15 mmol), nitromethane (10 equiv., 1.5 mmol), (*S*)-**Cu2** (10 mol%), and Ag_2_O (5 mol%) in 0.5 mL solvent were stirred for 24 h at RT. *^b^* The yields were determined by the ^1^H NMR analysis of the crude mixture. *^c^* 30 equiv. of CH_3_NO_2_ was used. *^d^* The racemic **2a** was obtained. DCE = 1,2-dichloroethane. THF = tetrahydrofuran.

**Table 3:** The optimization of the nitroaldol reaction of **1a** and control experiments *^a^*.

Entry	Catalyst (*x* mol%)	Additive (*x* mol%)	T, °C	Yield of 2a (%) *^b^*	Yield of 3a (%) *^b^*	Yield of 4a (%) *^b^*
1	(*S*)-**Cu2** (10)	Ag_2_O (5)	50	4	81	14
2	(*S*)-**Cu2** (10)	Ag_2_O (5)	70	<1	81	18
3	(*S*)-**Cu2** (5)	Ag_2_O (2.5)	70	2	87	10
4	(*S*)-**Cu2** (2)	Ag_2_O (1)	70	4	87	8
5 *^c^*	(*S*)-**Cu2** (2)	Ag_2_O (1)	70	3	88	6
6	(*S*)-**Cu3**	NaOAc (10)	RT	11	–	–
7	(*S*)-**Cu3**	Ag_2_O (5)	50	7	78	13
8	(*S*)-**Cu3**	Ag_2_O (5)	70	6	75	17
9 *^c^*	CuCl_2_ + 1,10-phen (2)	Ag_2_O (1)	70	33	23	4
10	–	Ag_2_O (5)	50	NR	–	–
11	–	*t*BuOK (5)	50	85	–	–

*^a^* Reaction conditions: *o*-nitrobenzaldehyde **1a** (0.15 mmol), nitromethane (10 eq., 1.5 mmol), catalyst (*x* mol%), and an additive (*x* mol%) in 0.5 mL DCE were stirred for 24 h. *^b^* The yields were determined by the ^1^H NMR analysis of the crude mixture. *^c^* 5.0 equiv. of CH_3_NO_2_ was used. NR = no reaction.

## Data Availability

Data from the research described in the manuscript are available from the authors.

## Supplementary Materials

The following supporting information can be downloaded at: https://www.mdpi.com/article/10.3390/molecules29215207/s1, ^1^H, ^13^C, and ^19^F NMR spectra for all compounds, and HPLC traces of the products can be downloaded at: https://www.mdpi.com/article/10.3390/molecules29215207/s1.
