# Supplementary material for: Selectivity Control in Nitroaldol (Henry) Reaction by Changing the Basic Anion in a Chiral Copper(II) Complex Based on (S)-2-Aminomethylpyrrolidine and 3,5-Di-tert-butylsalicylaldehyde
Source: Molecules. 2024 Nov 4;29(21):5207. doi: 10.3390/molecules29215207 (PMC11547561; doi:10.3390/molecules29215207)

SUPPORTING INFORMATION

Selectivity Control in Nitroaldol (Henry) Reaction by Changing the Basic Anion in a Chiral Copper(II) Complex Based on (*S*)-2-Aminomethylpyrrolidine and 3,5-Di-*tert*-butylsalicylaldehyde

Olga V. Khromova [1], Lidiya V. Yashkina [1], Nadezhda V. Stoletova [1], Victor I. Maleev [1], Yuri N. Belokon [1,*] and Vladimir A. Larionov [1,2,*]

[1] A.N. Nesmeyanov Institute of Organoelement Compounds of Russian Academy of Sciences (INEOS RAS), Vavilov Str. 28, bld. 1, 119334 Moscow, Russian Federation
[2] Peoples' Friendship University of Russia (RUDN University), Miklukho-Maklaya Str. 6, 117198 Moscow, Russian Federation

*Correspondence: yubel@ineos.ac.ru (Y.N.B.), larionov@ineos.ac.ru (V.A.L.)

Table of Contents

^{1}H, ^{13}C and ^{19}F NMR spectra of the compounds 2

HPLC traces of the chiral compounds 11

^{1}H, ^{13}C and ^{19}F NMR spectra of the compounds

Figure S1. ^{1}H NMR spectrum of **2b** in $CDCl_3$.

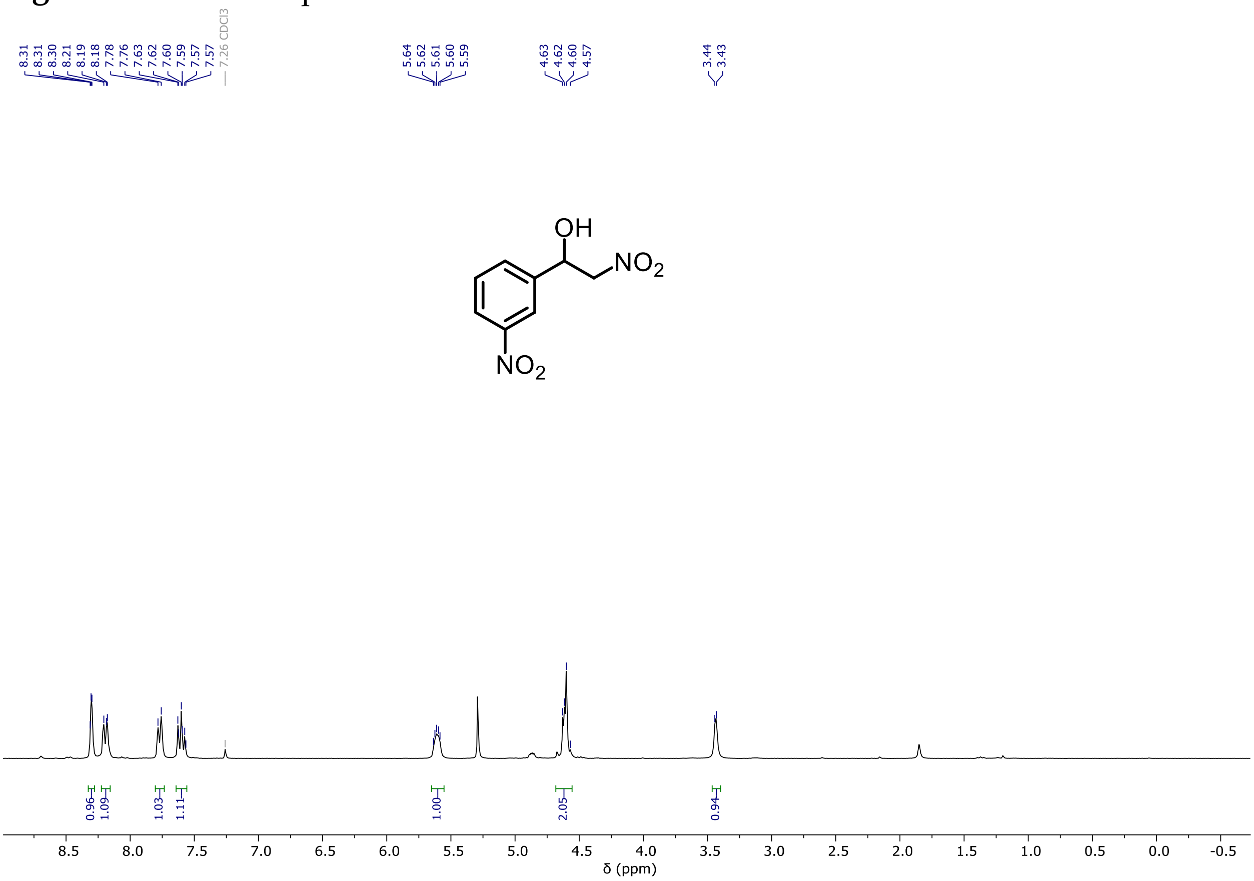

Figure S2. ^{1}H NMR spectrum of **2c** in $CDCl_3$.

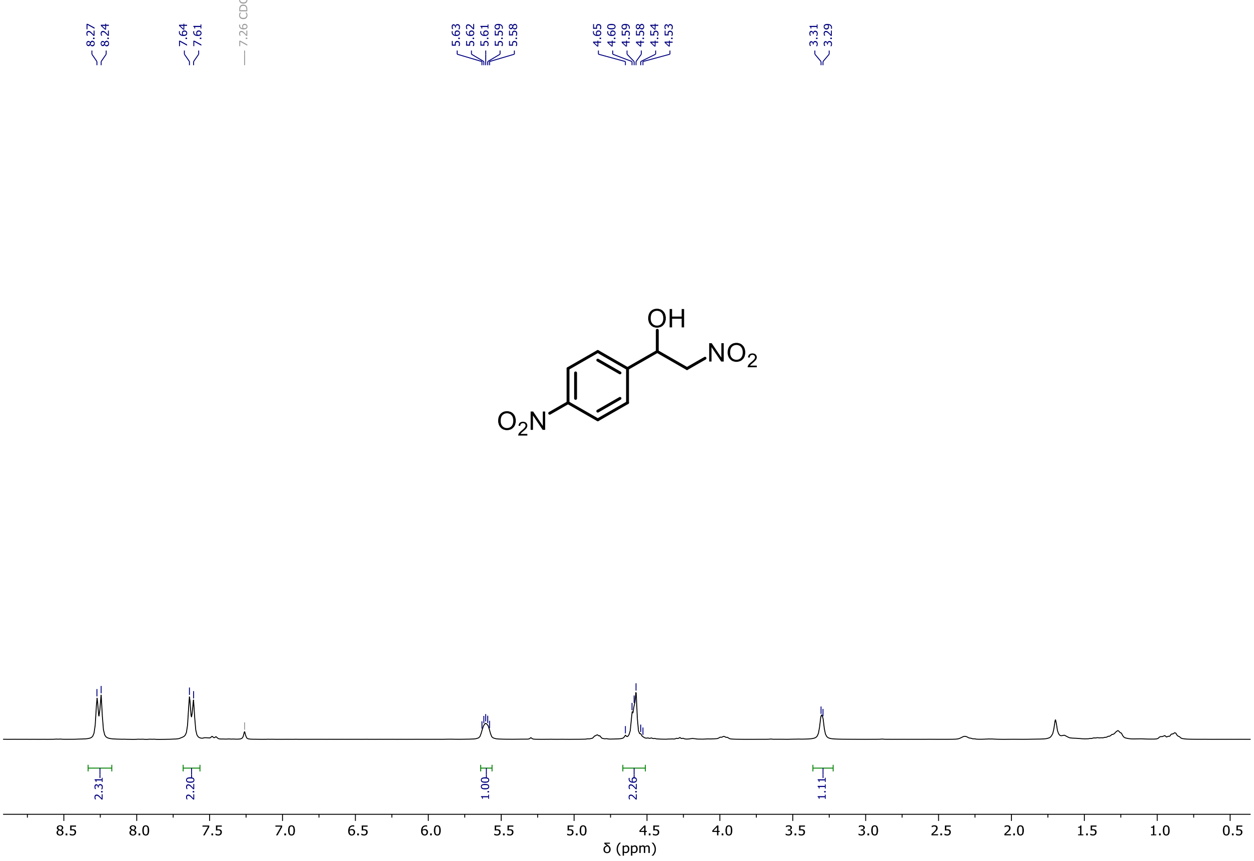

Figure S3. ^{1}H and ^{19}F NMR spectra of **2d** in $CDCl_3$.

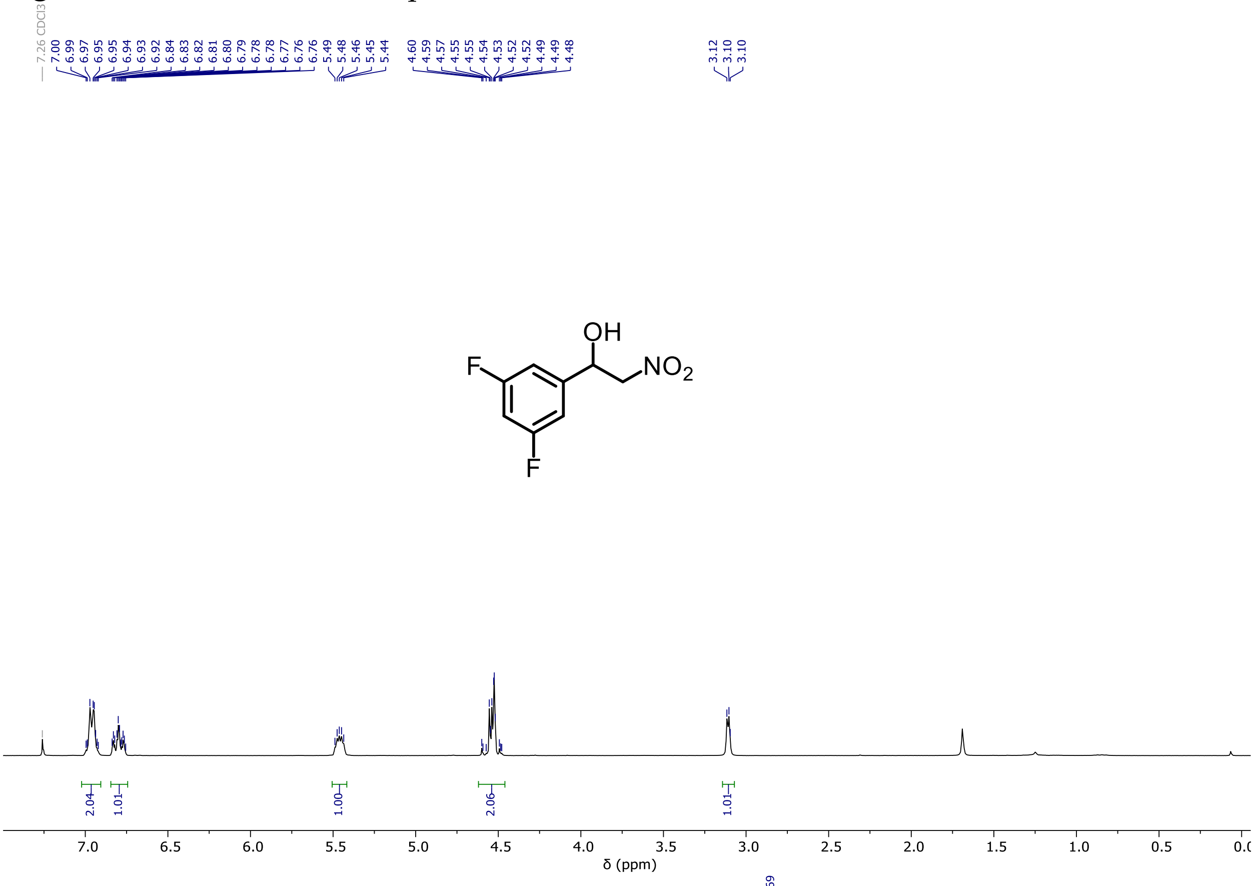

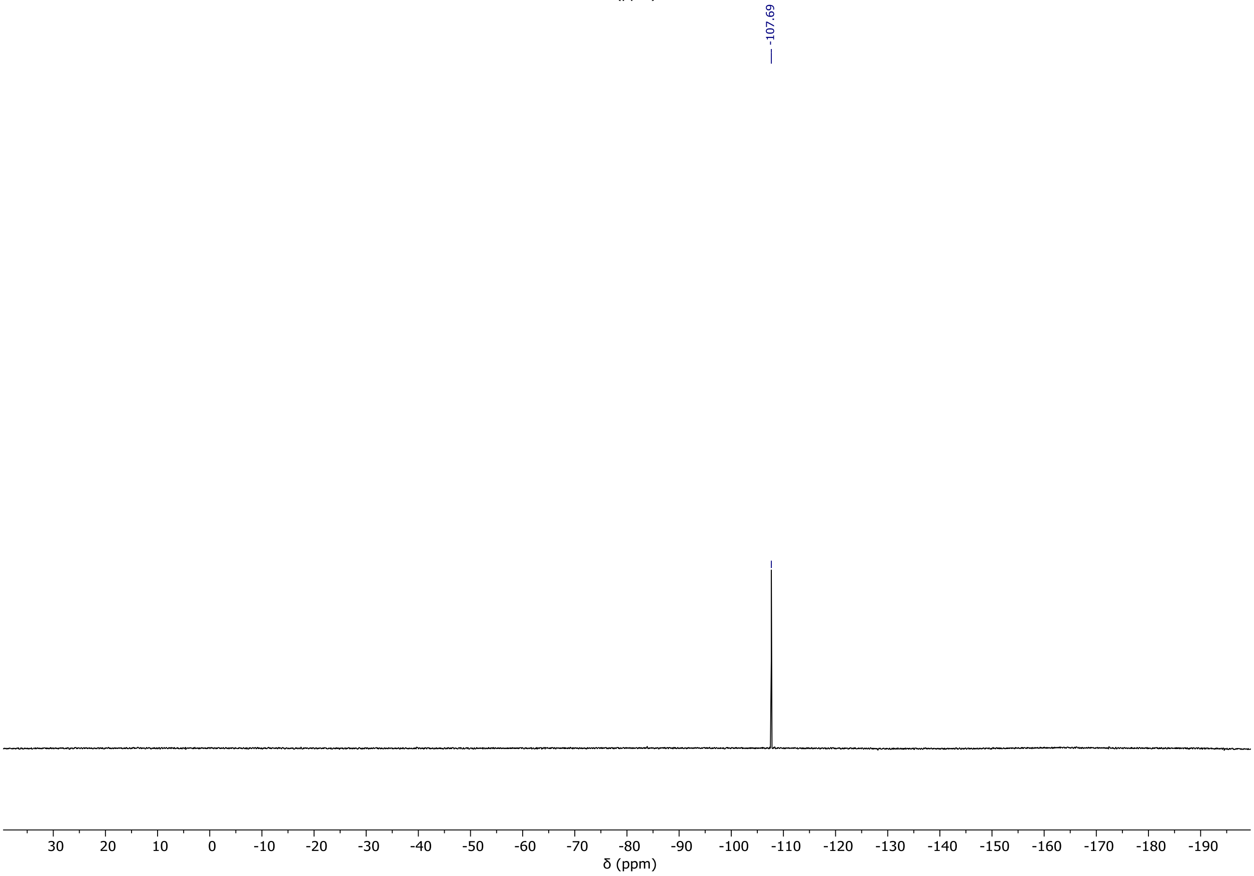

Figure S4. ^{1}H NMR spectrum of **2e** in $CDCl_3$.

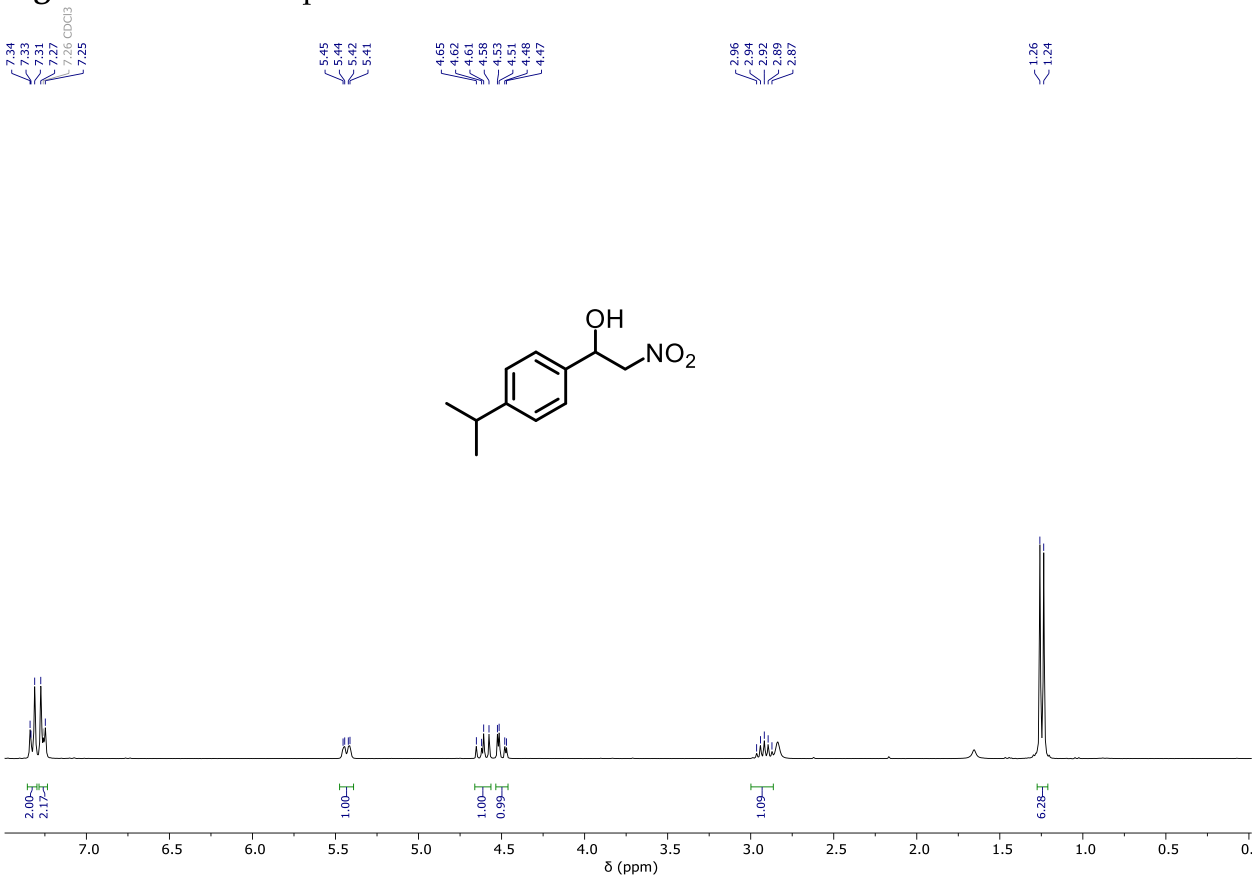

Figure S5. ^{1}H and ^{19}F NMR spectra of **2f** in $CDCl_3$.

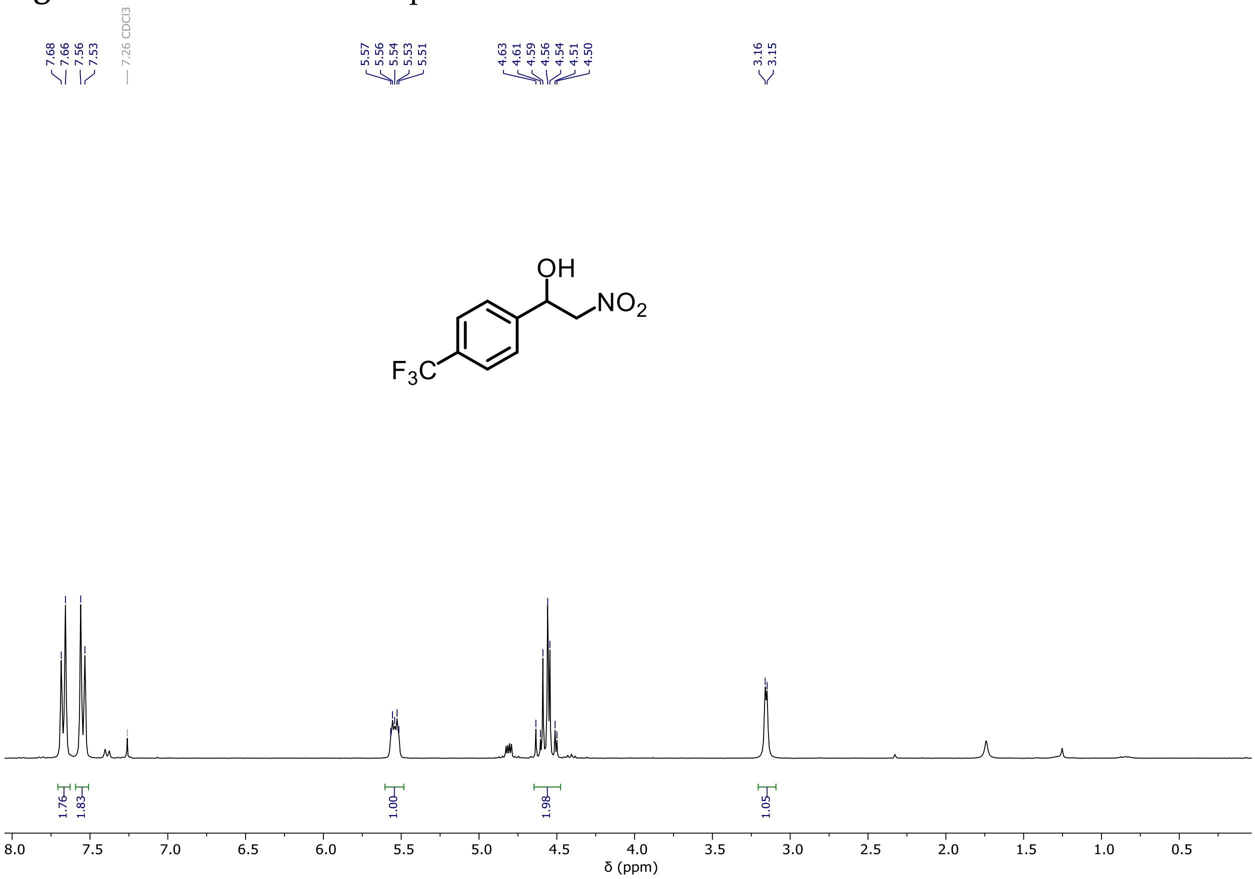

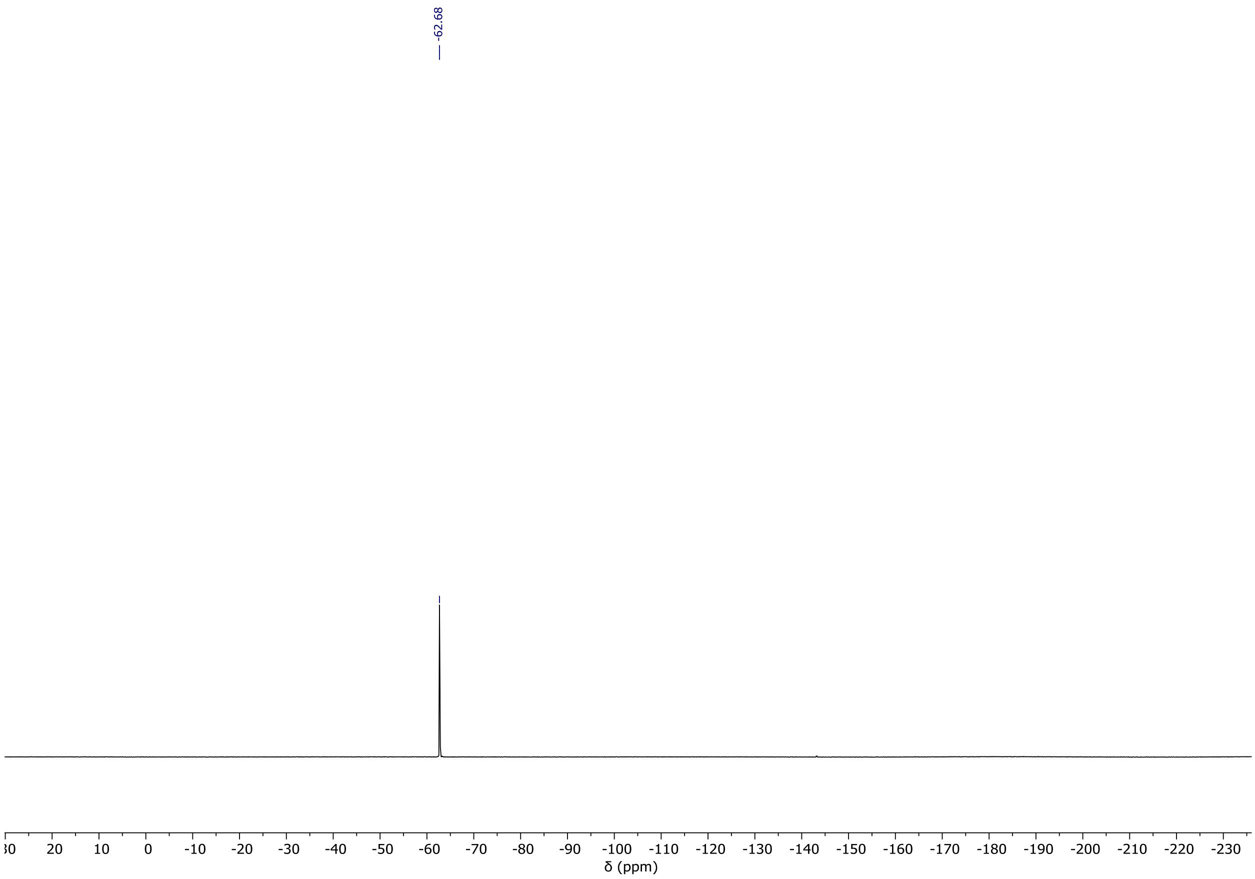

Figure S6. ^{1}H NMR spectrum of **2g** in $CDCl_3$.

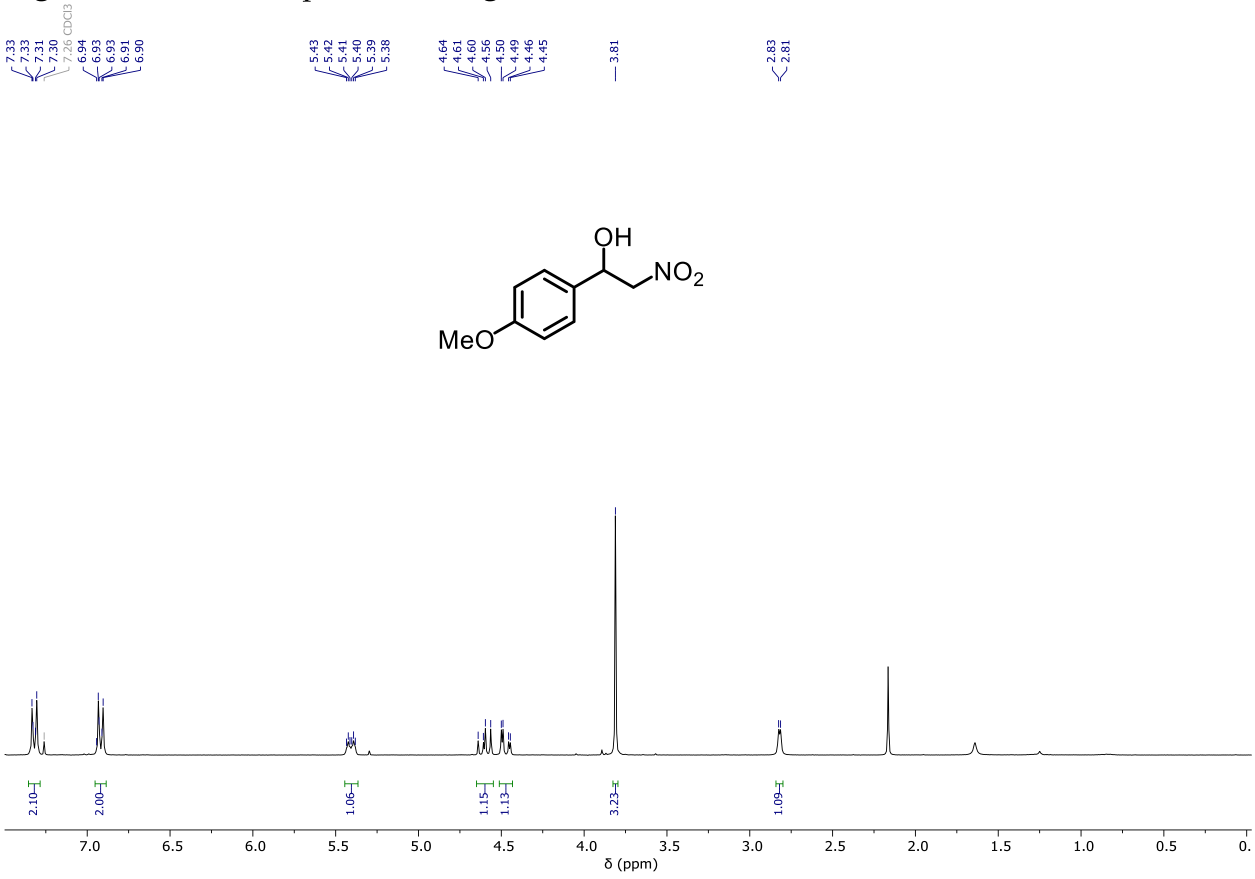

Figure S7. ^{1}H NMR spectrum of **2h** in $CDCl_3$.

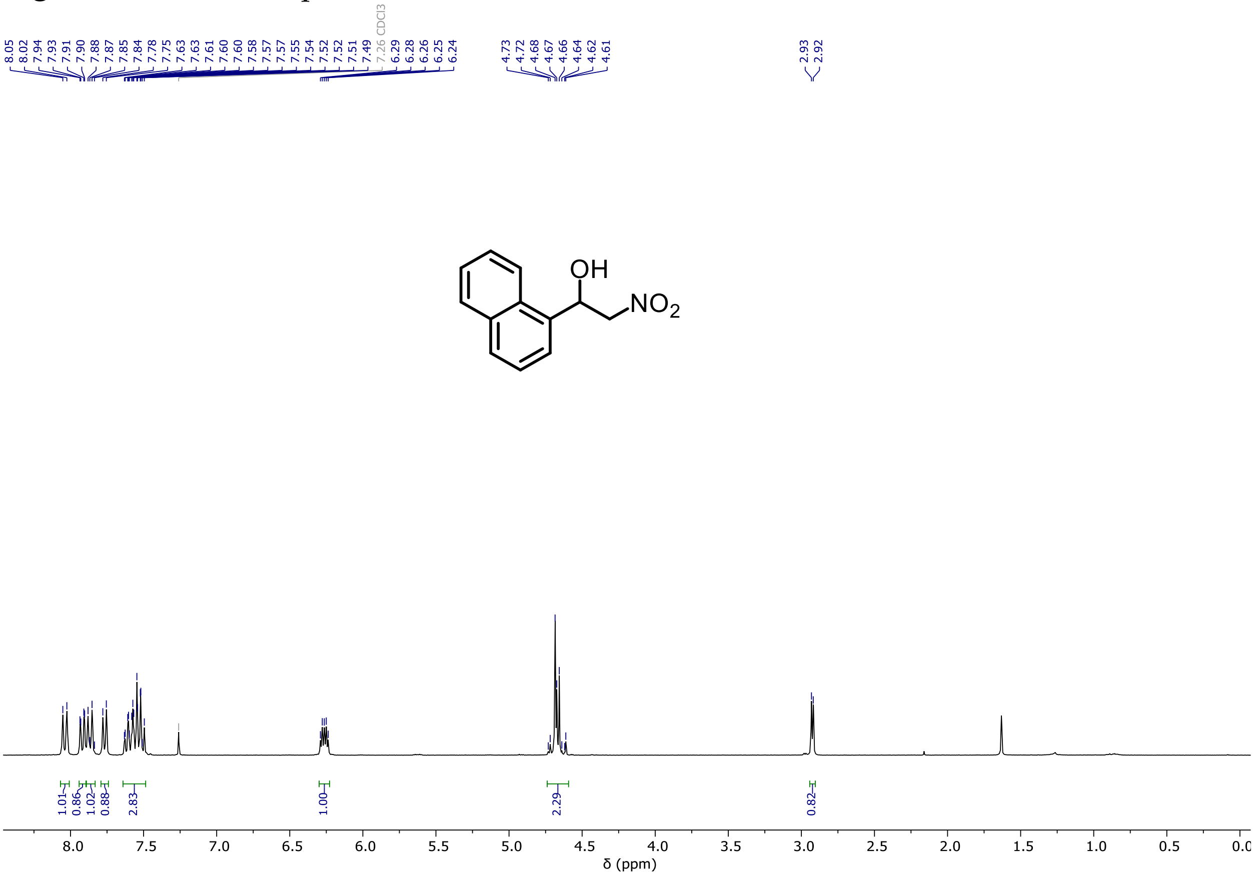

Figure S8. ^{1}H NMR spectrum of **2i** in $CDCl_3$.

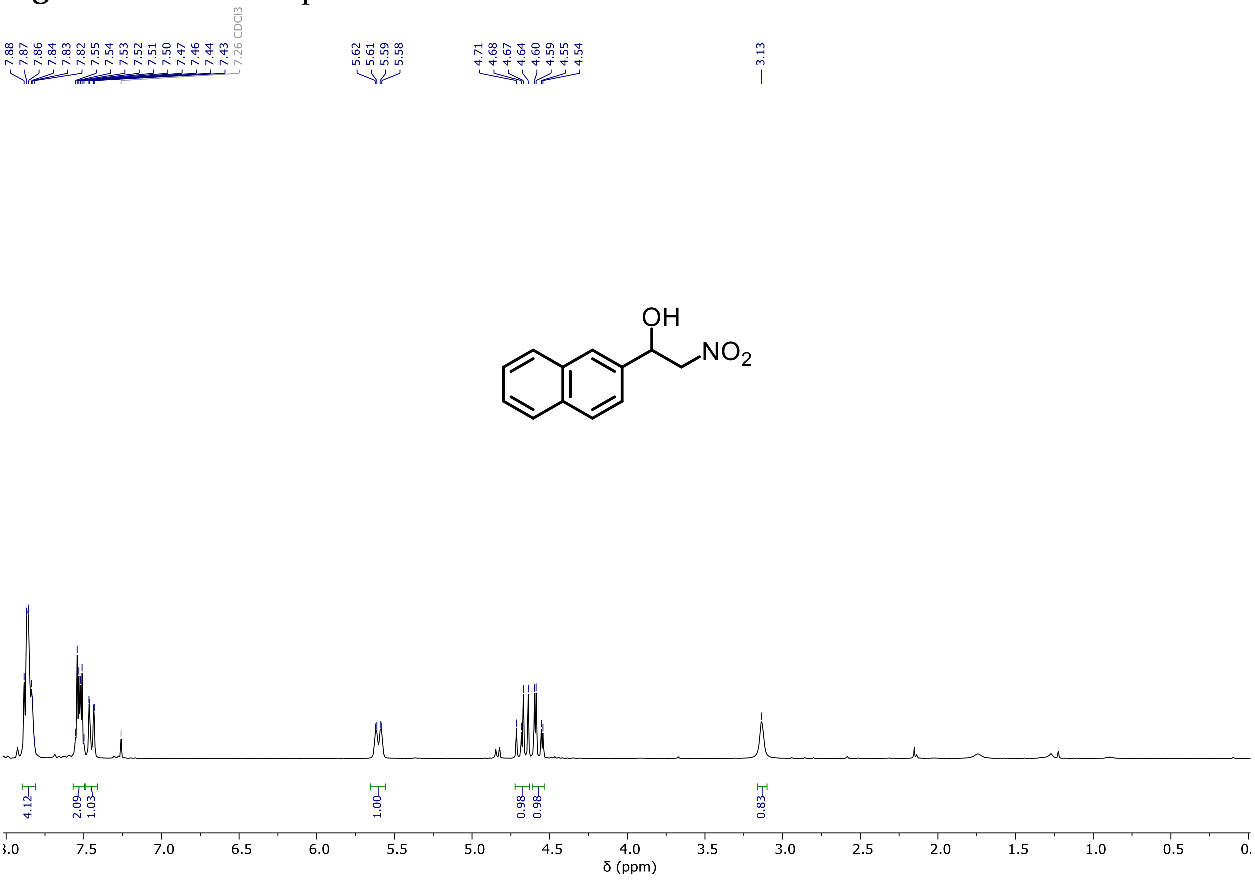

Figure S9. ^{1}H NMR spectrum of **3a** in $CDCl_3$.

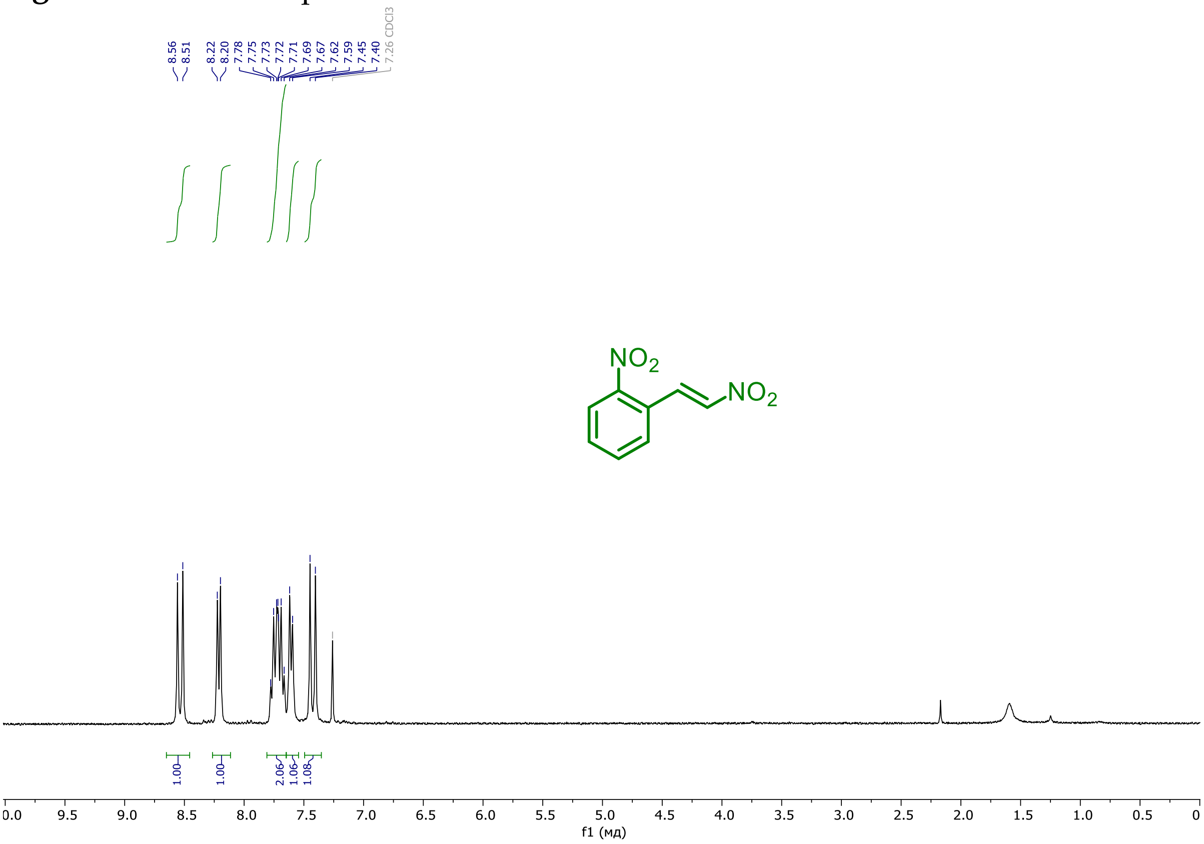

Figure S10. ^{1}H NMR spectrum of **3b** in $CDCl_3$.

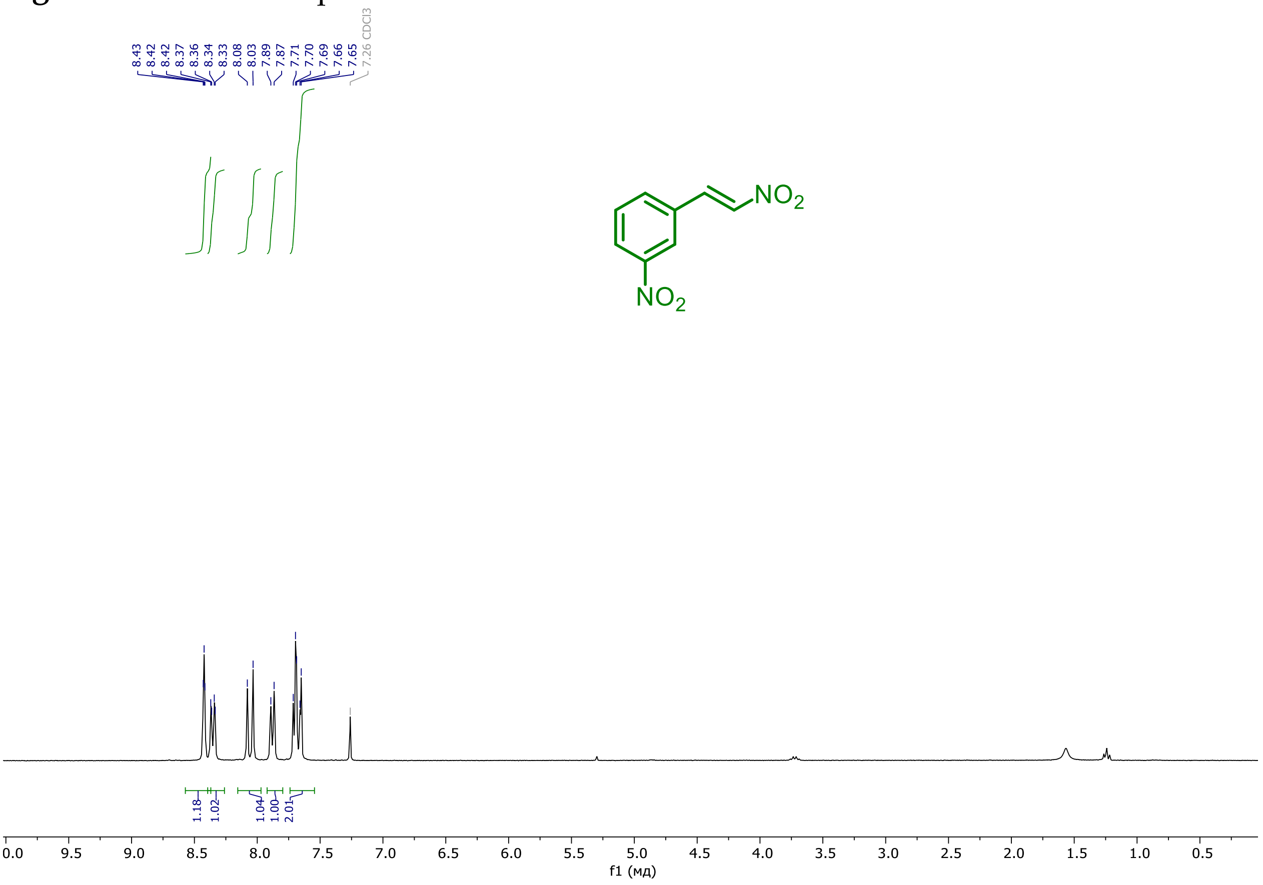

Figure S11. ^{1}H NMR spectrum of **3c** in acetone-d$_6$.

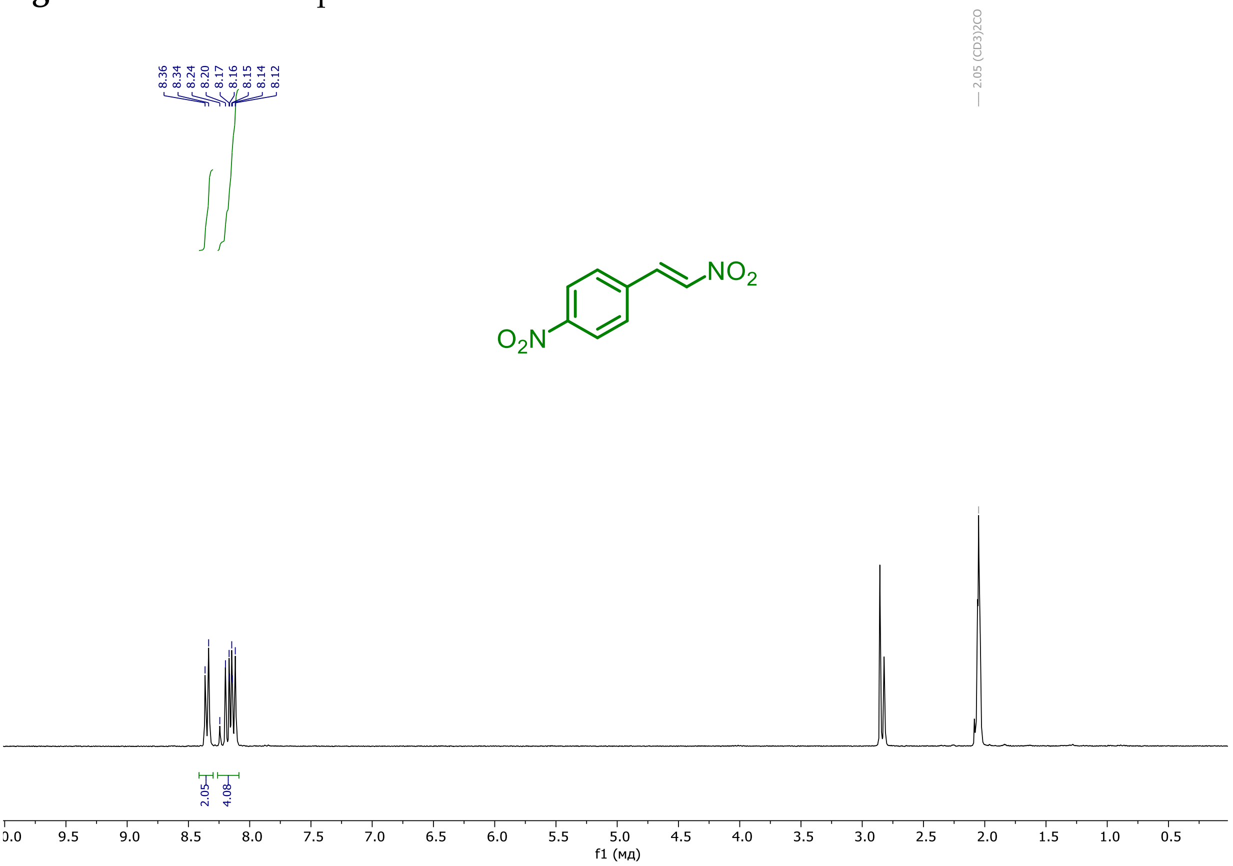

Figure S12. ^{1}H and ^{13}C NMR spectra of **3d** in CDCl$_3$.

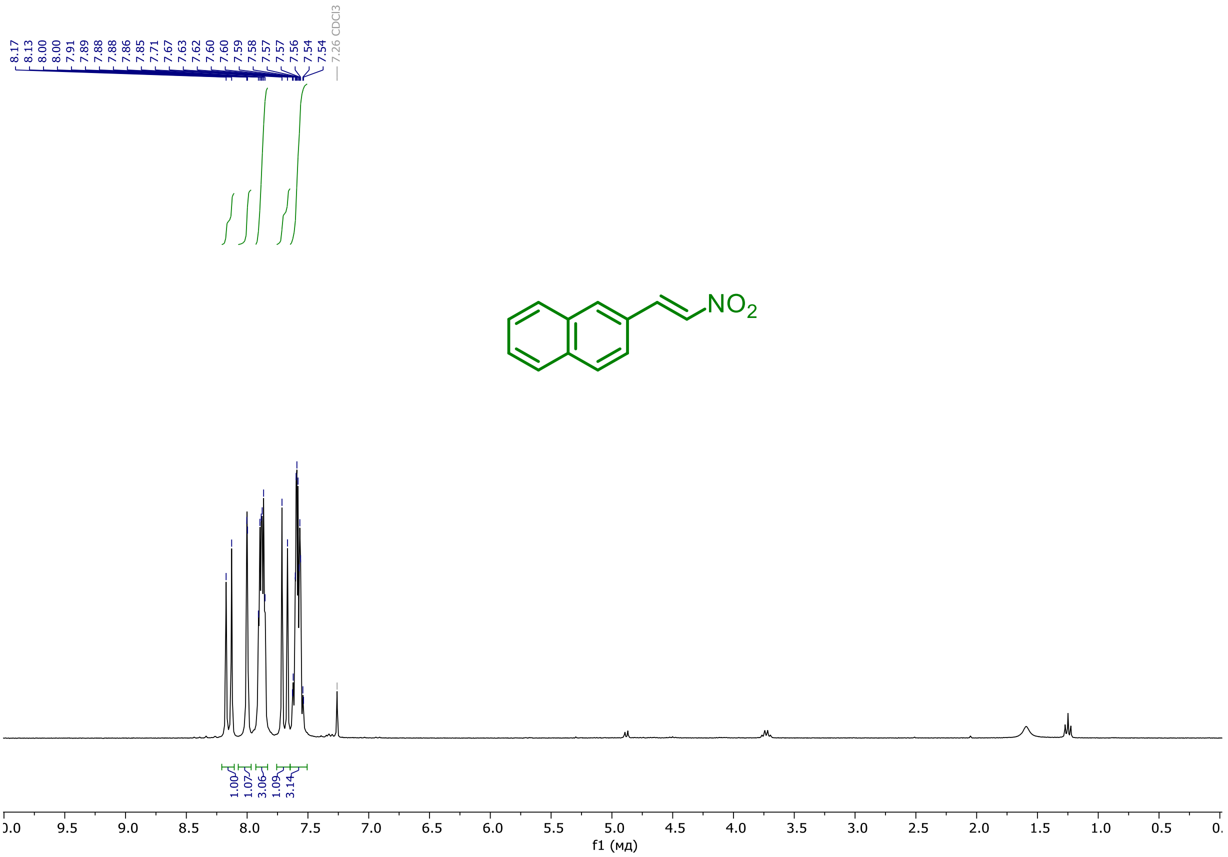

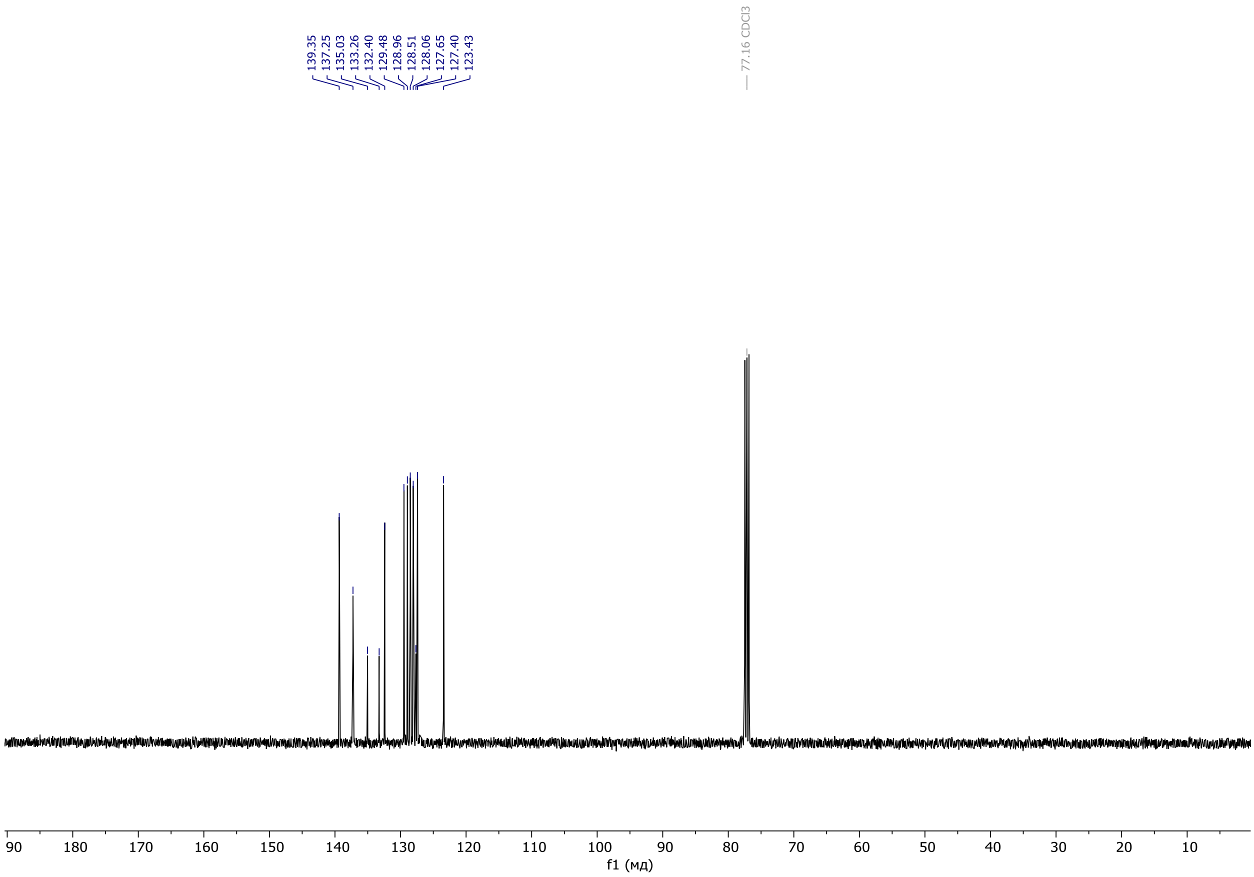

Figure S13. ^{1}H and ^{13}C NMR spectra of **3e** in $CDCl_3$.

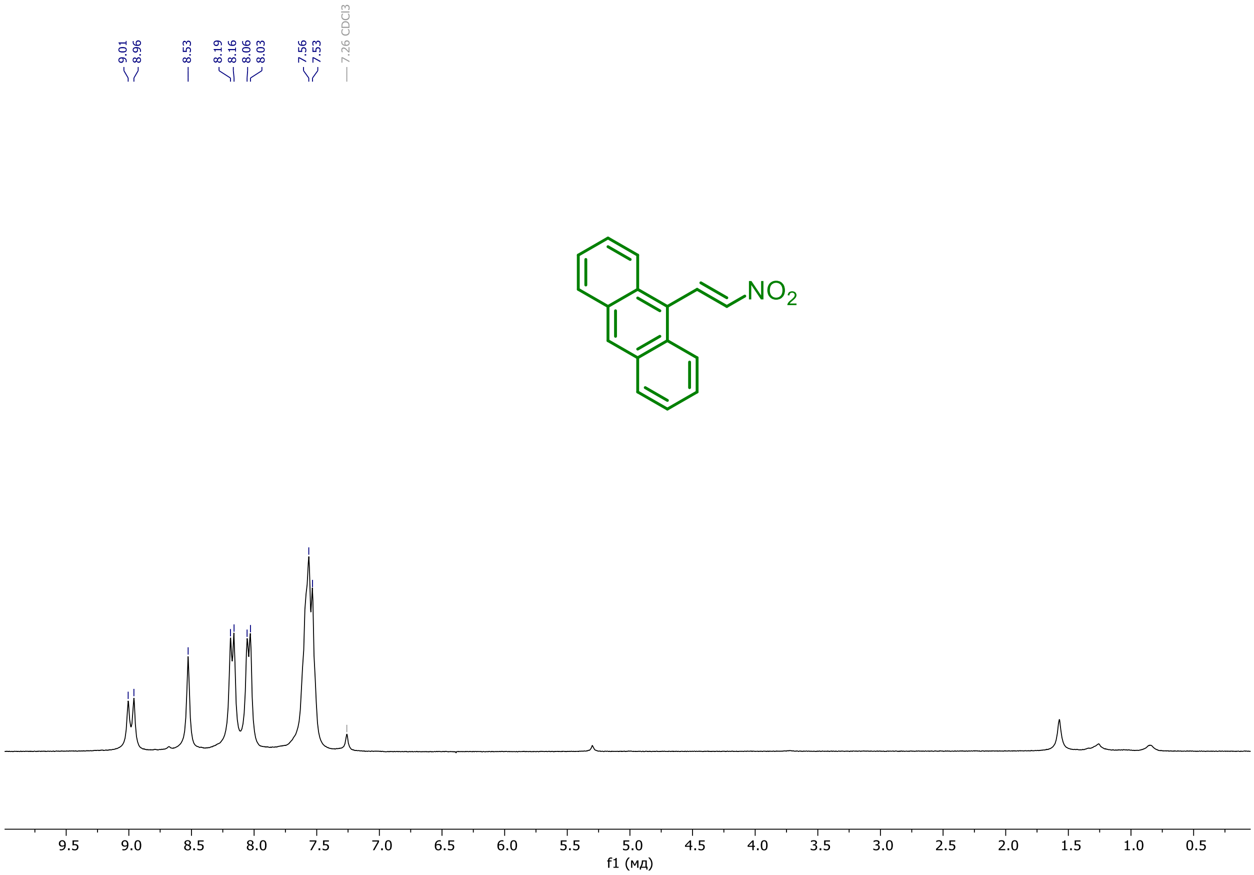

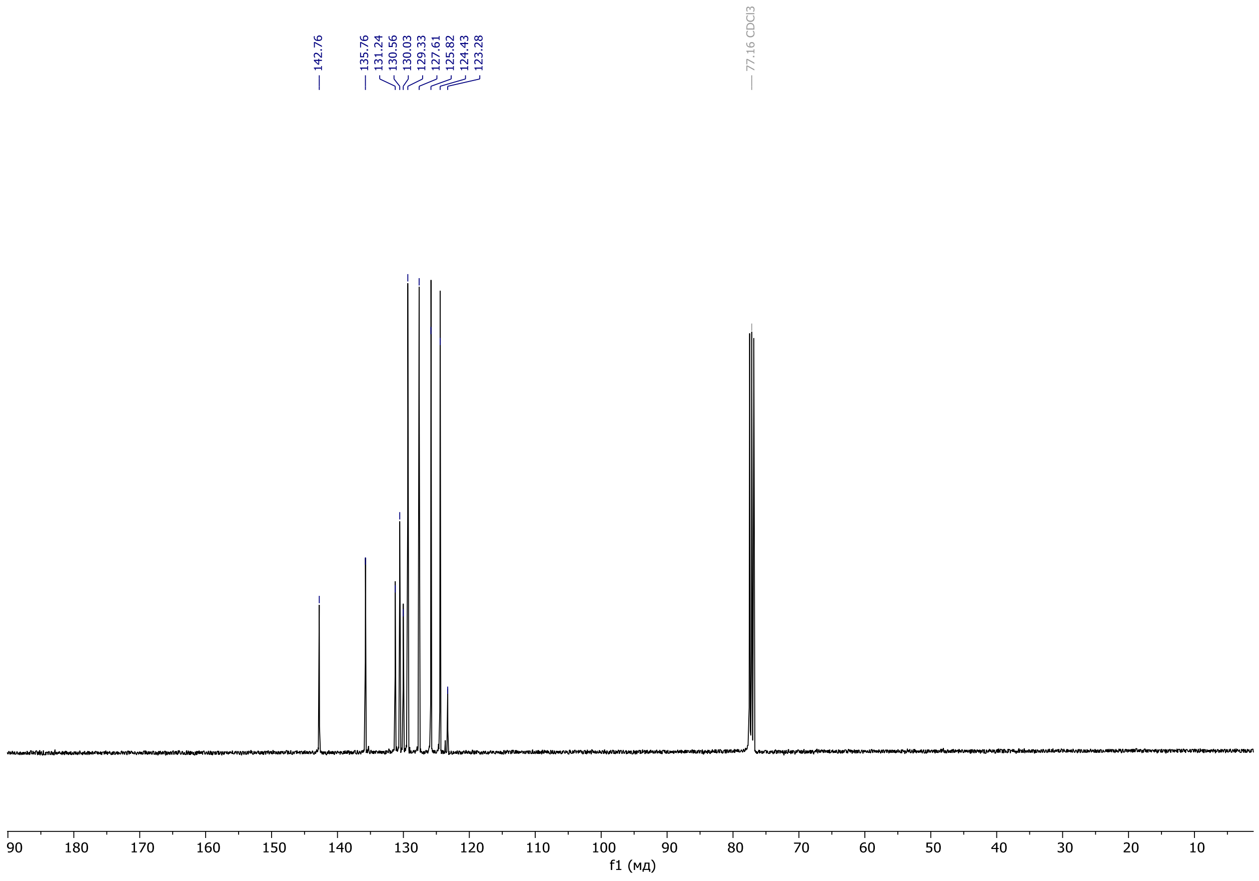

142.76
135.76
131.24
130.56
130.03
129.33
127.61
125.82
124.43
123.28
77.16 CDCl3
90
180
170
160
150
140
130
120
110
100
90
80
70
60
50
40
30
20
10
f1 (мд)

HPLC traces of the chiral compounds

Figure S14. HPLC traces of **2b**.

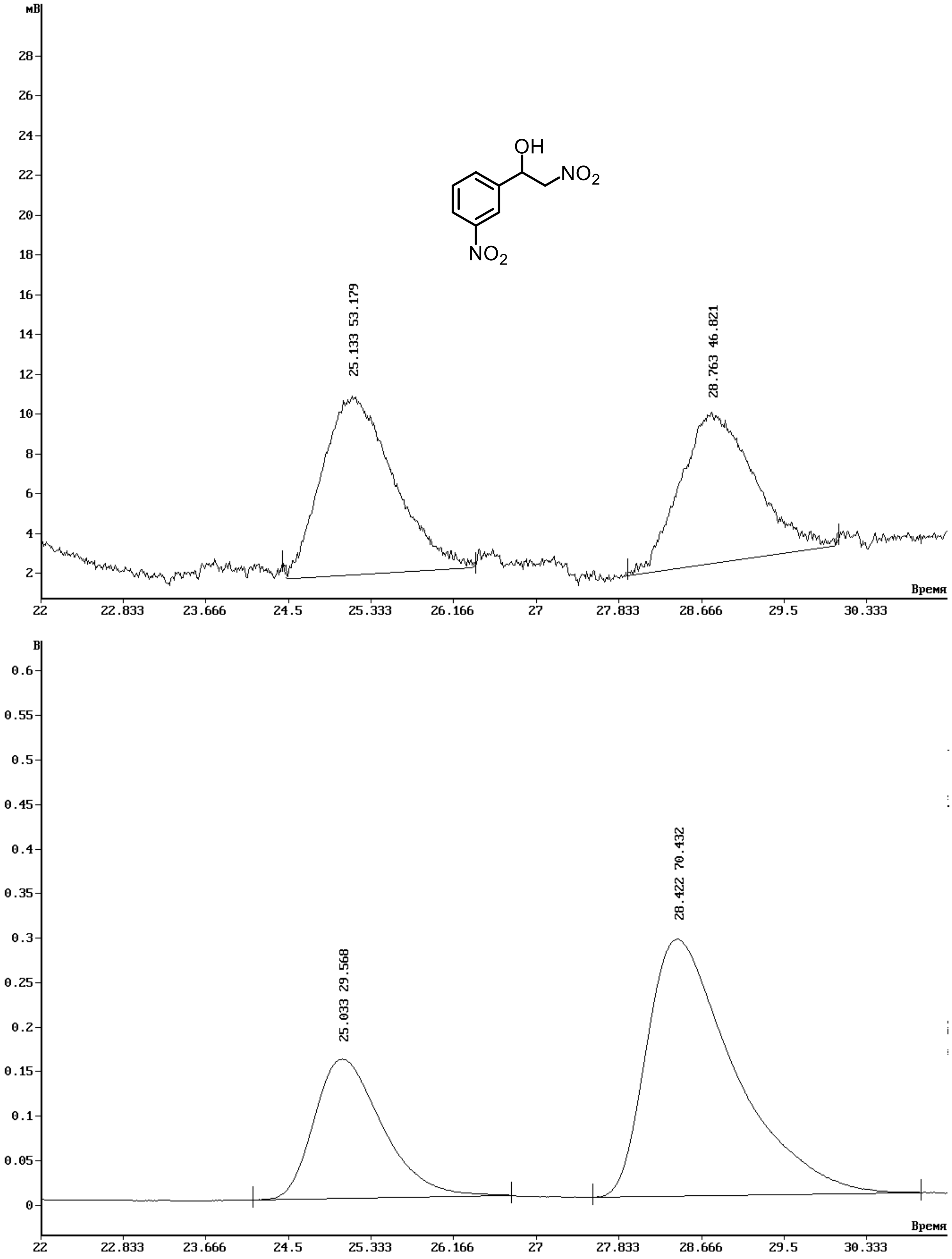

Figure S15. HPLC traces of **2c**.

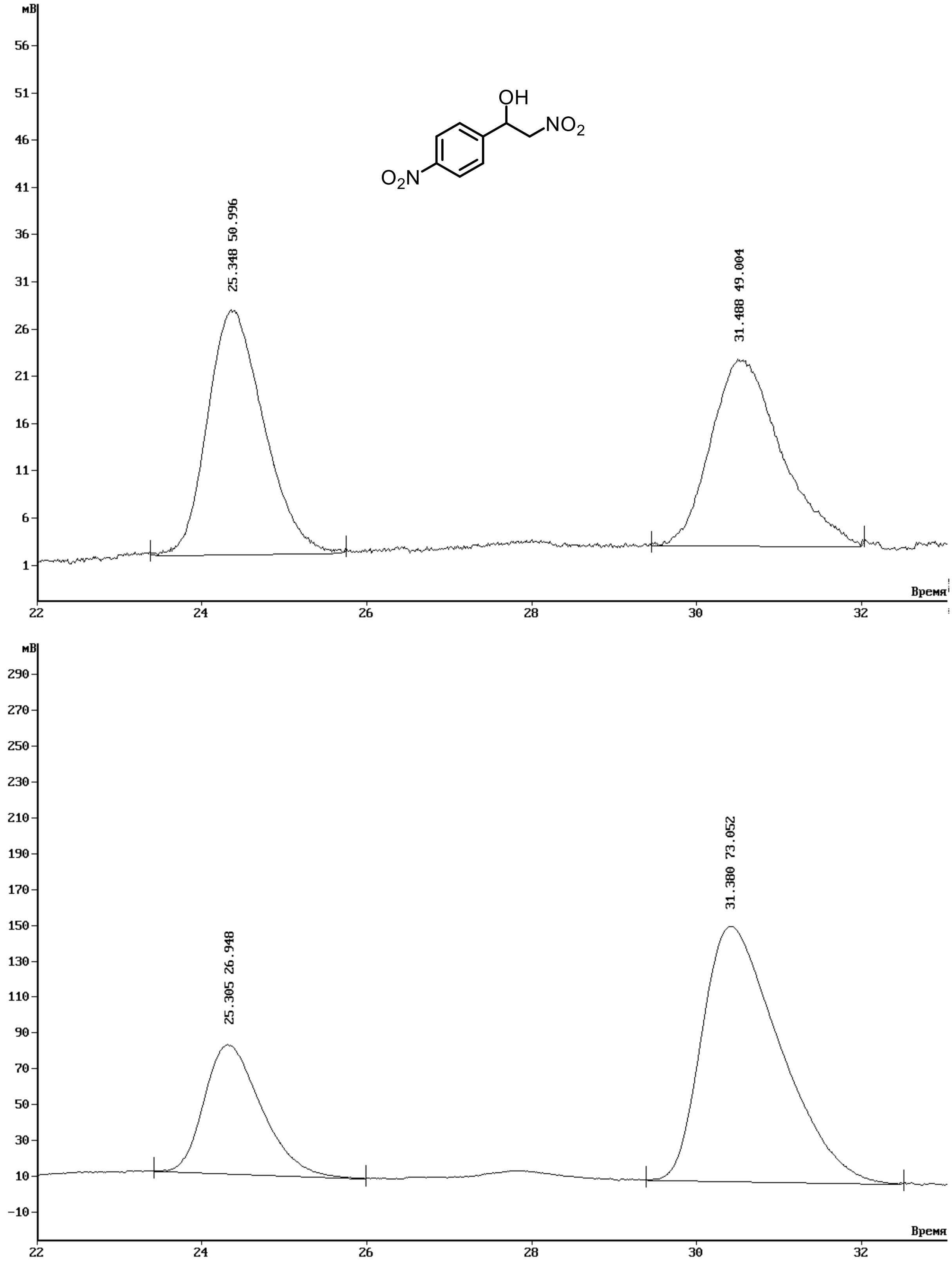

Figure S16. HPLC traces of **2d**.

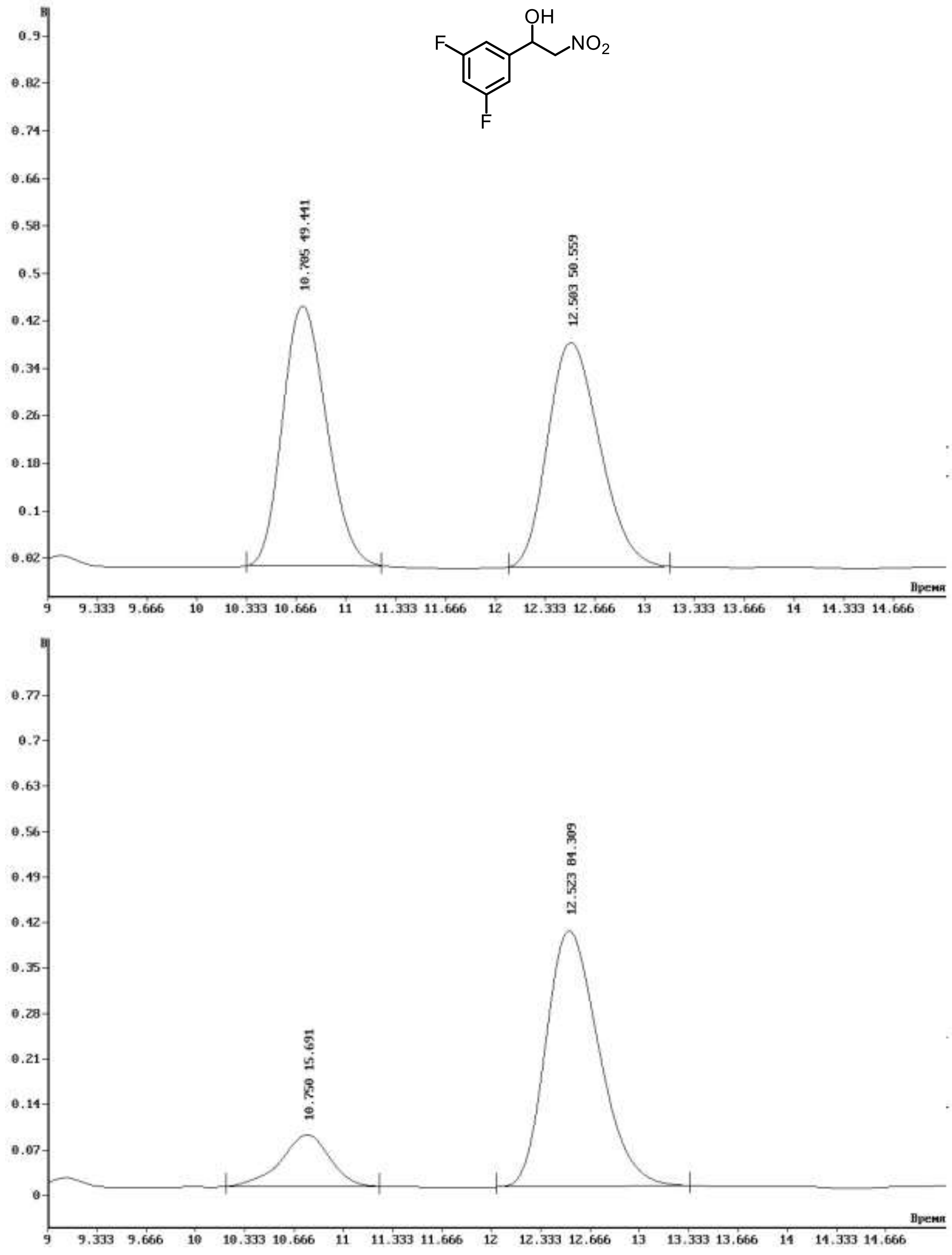

Figure S17. HPLC traces of **2e**.

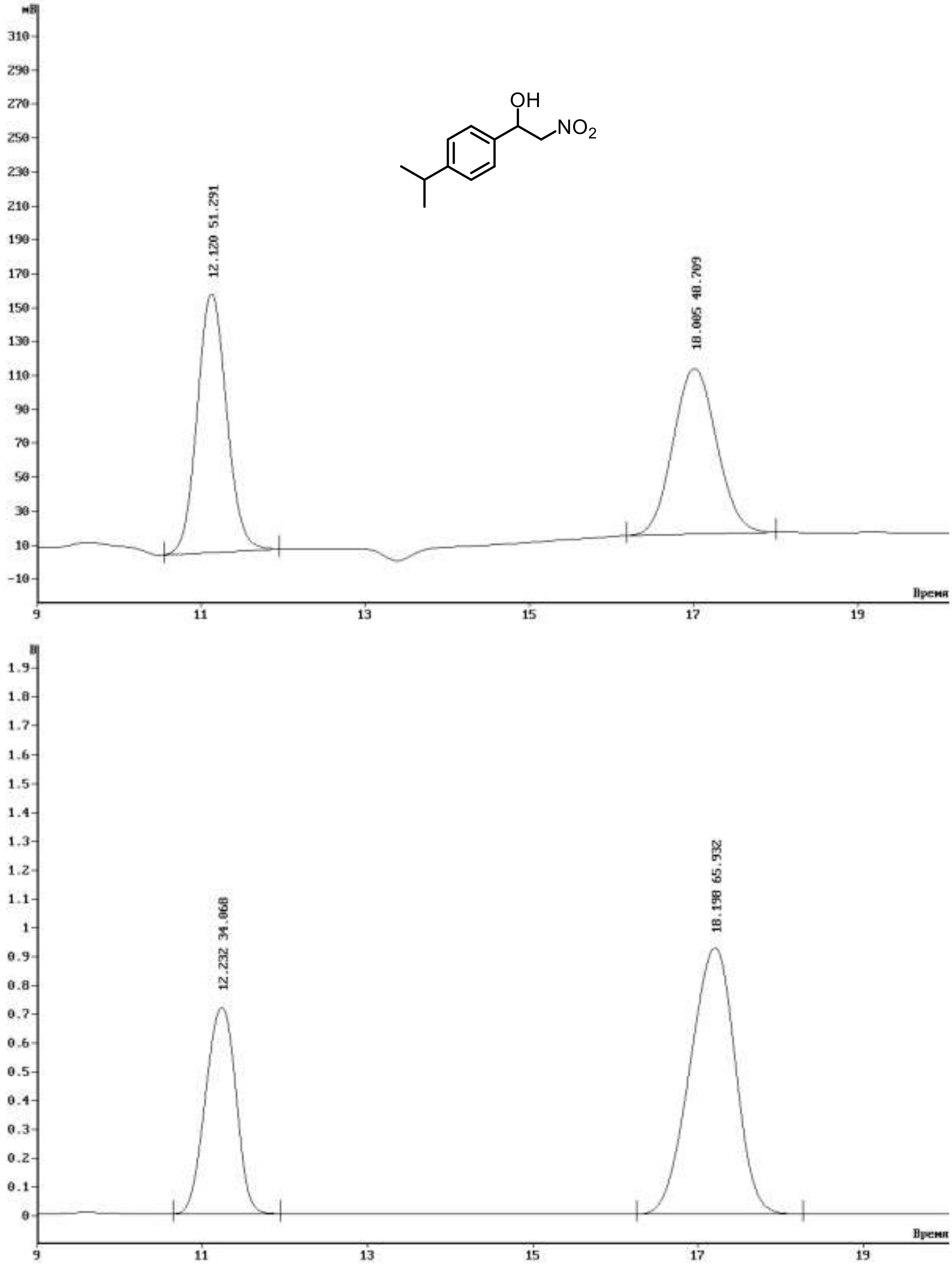

Figure S18. HPLC traces of **2f**.

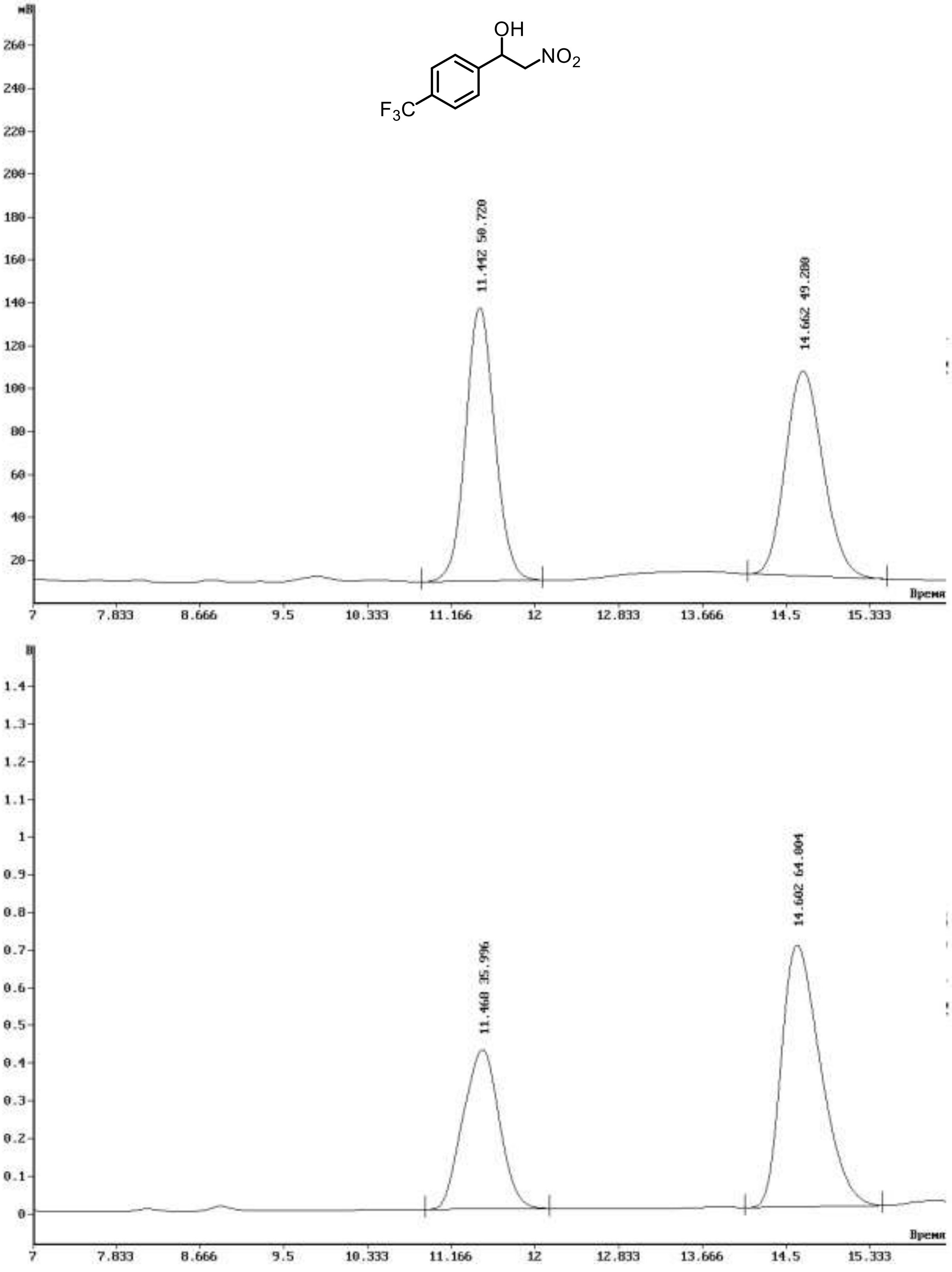

Figure S19. HPLC traces of **2g**.

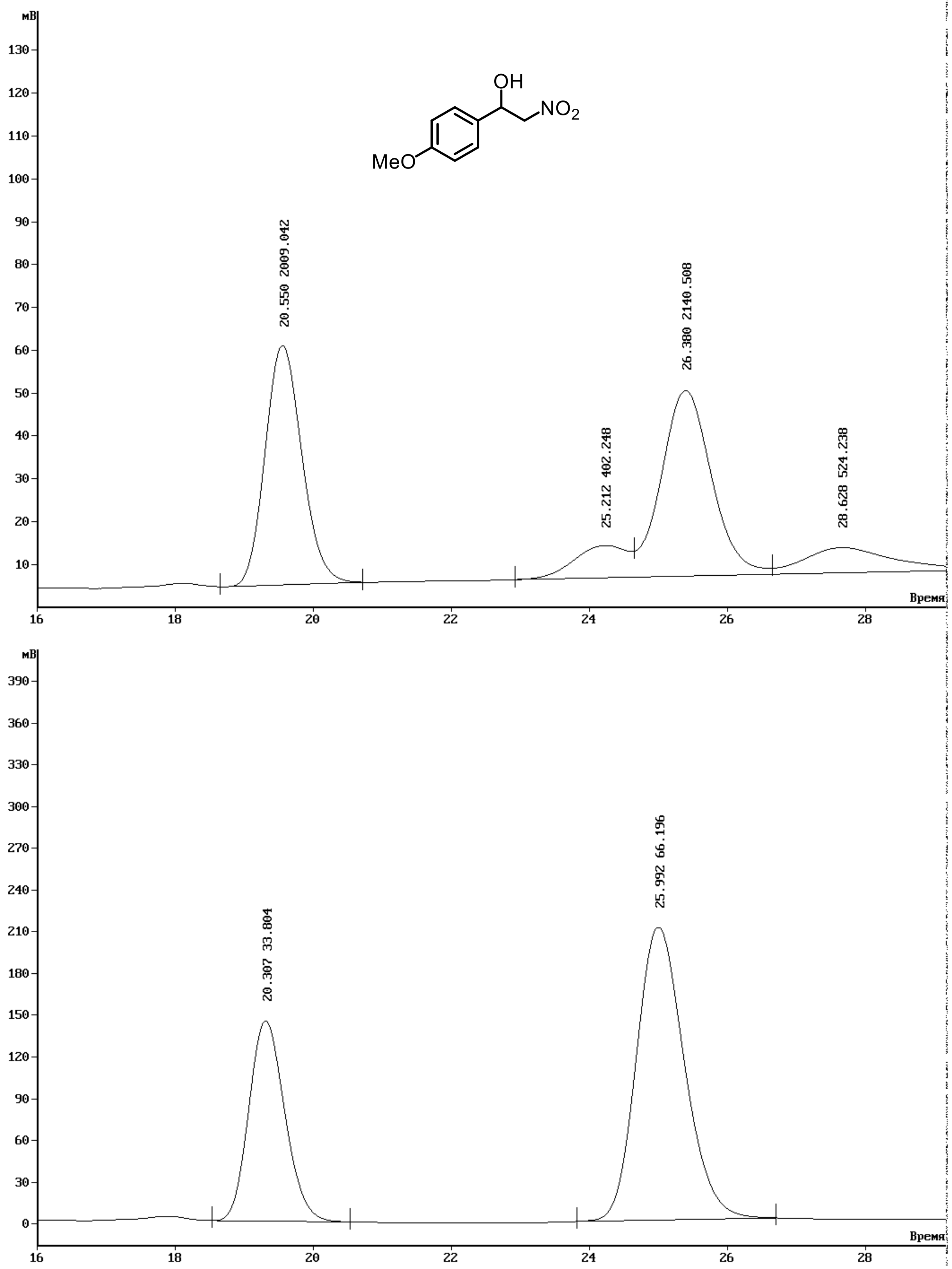

Figure S20. HPLC traces of **2h**. t_R(major) = 24.8 min, t_R(minor) = 18.0 min); area%(major) = 82%, area%(minor) = 18%.

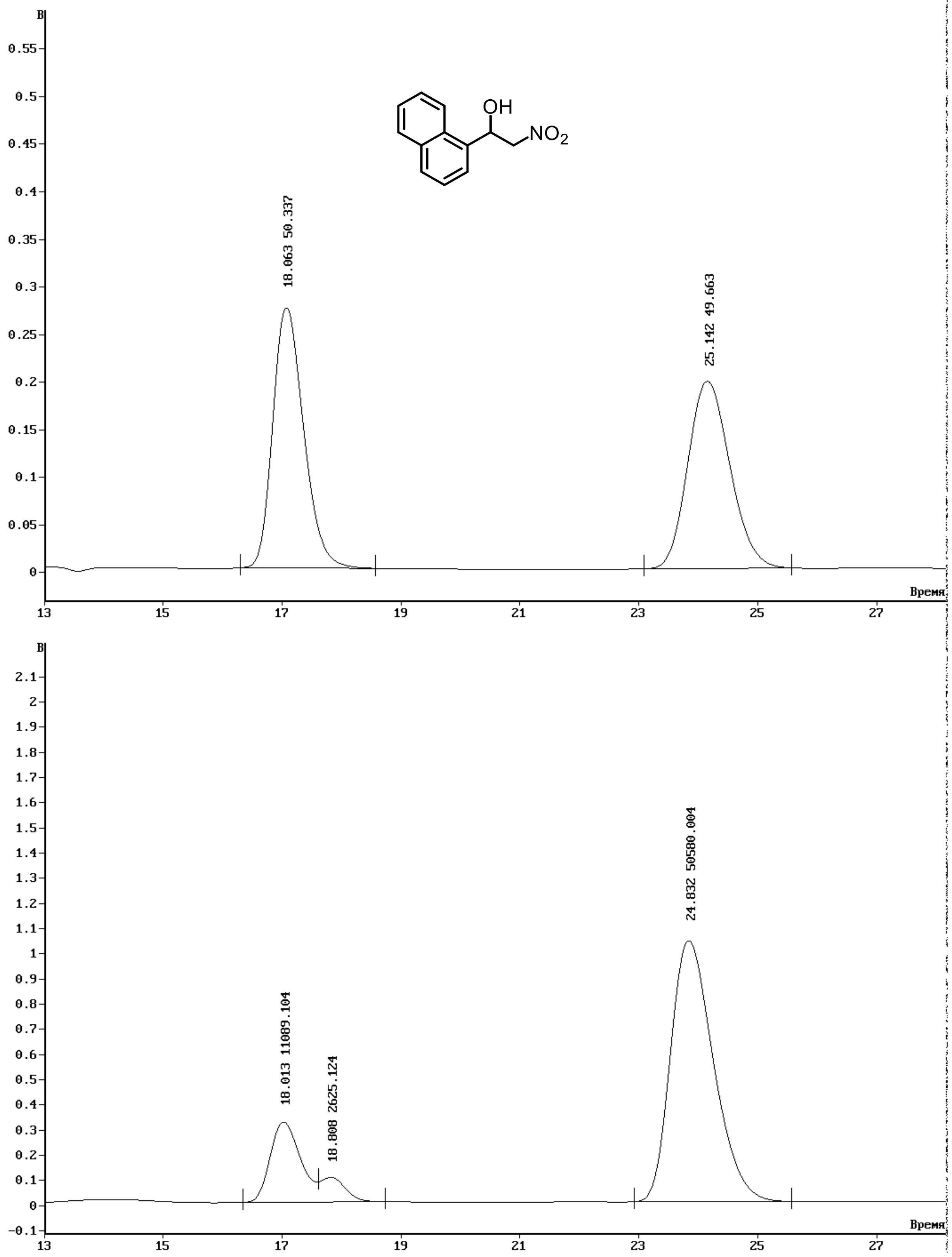

Figure S21. HPLC traces of **2i**.

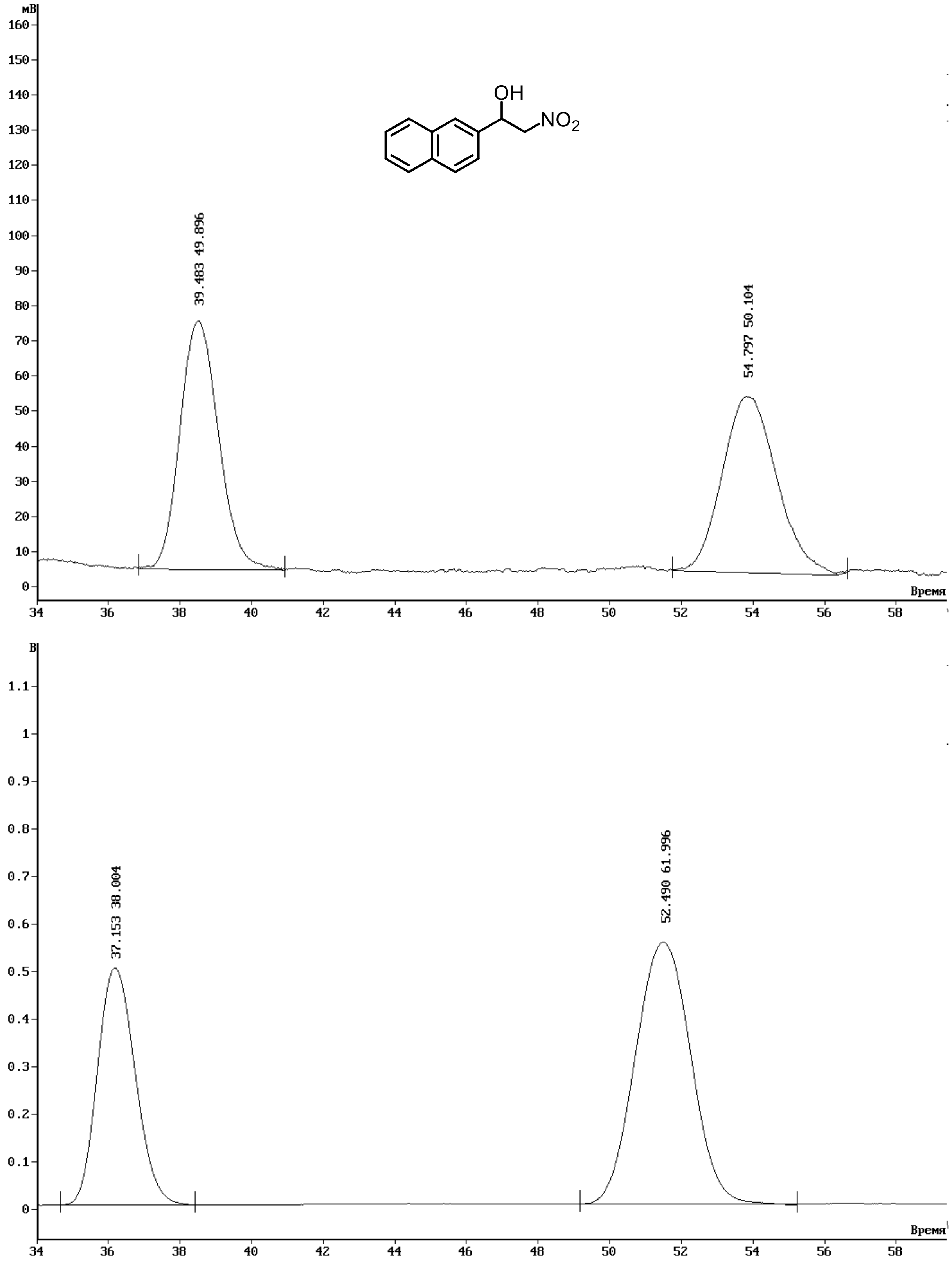

Supplement: Supplementary file 1 [file molecules-29-05207-s001.zip › molecules-3269424-supplementary.pdf]
